# Lung Cancer Screening Before and After a Multifaceted Electronic Health Record Intervention: A Nonrandomized Controlled Trial

**DOI:** 10.1001/jamanetworkopen.2024.15383

**Published:** 2024-06-07

**Authors:** Polina V. Kukhareva, Haojia Li, Tanner J. Caverly, Angela Fagerlin, Guilherme Del Fiol, Rachel Hess, Yue Zhang, Jorie M. Butler, Chelsey Schlechter, Michael C. Flynn, Chakravarthy Reddy, Joshua Choi, Christian Balbin, Isaac A. Warner, Phillip B. Warner, Claude Nanjo, Kensaku Kawamoto,

**Affiliations:** 1 Department of Biomedical Informatics, University of Utah, Salt Lake City; 2 Study Design and Biostatistics Center, University of Utah, Salt Lake City; 3 Center for Clinical Management Research, Department of Veterans Affairs, Ann Arbor, Michigan; 4 Department of Learning Health Sciences, University of Michigan, Ann Arbor; 5 Department of Internal Medicine, University of Michigan, Ann Arbor; 6 Department of Population Health Sciences, University of Utah, Salt Lake City; 7 Salt Lake City VA Informatics Decision-Enhancement and Analytic Sciences Center for Innovation, Salt Lake City, Utah; 8 Department of Internal Medicine, University of Utah, Salt Lake City; 9 Geriatrics Research and Education Center, George E. Wahlen Department of Veterans Affairs Medical Center, Salt Lake City, Utah; 10 Department of Pediatrics, University of Utah, Salt Lake City; 11 Community Physicians Group, University of Utah Health, Salt Lake City

## Abstract

**Importance:**

Lung cancer is the deadliest cancer in the US. Early-stage lung cancer detection with lung cancer screening (LCS) through low-dose computed tomography (LDCT) improves outcomes.

**Objective:**

To assess the association of a multifaceted clinical decision support intervention with rates of identification and completion of recommended LCS-related services.

**Design, Setting, and Participants:**

This nonrandomized controlled trial used an interrupted time series design, including 3 study periods from August 24, 2019, to April 27, 2022: baseline (12 months), period 1 (11 months), and period 2 (9 months). Outcome changes were reported as shifts in the outcome level at the beginning of each period and changes in monthly trend (ie, slope). The study was conducted at primary care and pulmonary clinics at a health care system headquartered in Salt Lake City, Utah, among patients aged 55 to 80 years who had smoked 30 pack-years or more and were current smokers or had quit smoking in the past 15 years. Data were analyzed from September 2023 through February 2024.

**Interventions:**

Interventions in period 1 included clinician-facing preventive care reminders, an electronic health record–integrated shared decision-making tool, and narrative LCS guidance provided in the LDCT ordering screen. Interventions in period 2 included the same clinician-facing interventions and patient-facing reminders for LCS discussion and LCS.

**Main Outcome and Measure:**

The primary outcome was LCS care gap closure, defined as the identification and completion of recommended care services. LCS care gap closure could be achieved through LDCT completion, other chest CT completion, or LCS shared decision-making.

**Results:**

The study included 1865 patients (median [IQR] age, 64 [60-70] years; 759 female [40.7%]). The clinician-facing intervention (period 1) was not associated with changes in level but was associated with an increase in slope of 2.6 percentage points (95% CI, 2.4-2.7 percentage points) per month in care gap closure through any means and 1.6 percentage points (95% CI, 1.4-1.8 percentage points) per month in closure through LDCT. In period 2, introduction of patient-facing reminders was associated with an immediate increase in care gap closure (2.3 percentage points; 95% CI, 1.0-3.6 percentage points) and closure through LDCT (2.4 percentage points; 95% CI, 0.9-3.9 percentage points) but was not associated with an increase in slope. The overall care gap closure rate was 175 of 1104 patients (15.9%) at the end of the baseline period vs 588 of 1255 patients (46.9%) at the end of period 2.

**Conclusions and Relevance:**

In this study, a multifaceted intervention was associated with an improvement in LCS care gap closure.

**Trial Registration:**

ClinicalTrials.gov Identifier: NCT04498052

## Key Points

**Question:**

Is a multifaceted lung cancer screening intervention, including a shared decision-making tool, clinician-facing reminders, narrative guidance provided in the ordering process, and patient-facing reminders, associated with improved screening-related care (care gap closure) for lung cancer screening?

**Findings:**

In this nonrandomized controlled trial of 1865 patients aged 55 to 80 years who smoked 30 pack-years or more, the care gap closure rate for lung cancer screening improved from 15.9% to 46.9%.

**Meaning:**

This study found that a multifaceted electronic health record–integrated intervention was associated with increased care gap closure for lung cancer screening.

## Teaser

This nonrandomized controlled trial investigates the association of an electronic health record–based lung cancer screening intervention with improved screening-related care.

## Introduction

Lung cancer, the leading cause of cancer-related deaths in the US, continues to be a formidable health challenge.^1^ The US Preventive Services Task Force (USPSTF) recommends offering lung cancer screening (LCS) through low-dose computed tomography (LDCT) to individuals with a significant tobacco use history.^2,3^ Although LCS reduces mortality by as much as 20%,^4,5^ the LCS rate among eligible individuals was approximately 6.5% in 2020.^6^

To address low LCS rates, health care systems across the US are beginning to implement clinical decision support (CDS) interventions to support LCS.^7,8,9,10^ In a study at University of Utah Health (UUH), a multifaceted intervention, including clinician-facing reminders and an electronic health record (EHR)–integrated shared decision-making (SDM) tool, was associated with an increase in LDCT ordering (adjusted odds ratio [OR], 4.9, 95% CI, 3.4-6.9).^7,8^ Another study reported that implementing EHR prompts was associated with an increase in LDCT ordering (adjusted OR, 1.04; 95% CI, 1.01-1.07).^9^

These prior studies of EHR-integrated CDS interventions focused on LCS ordering and completion among patients who had not undergone LCS in the past year.^8,9^ While LDCT is central to meeting patient needs related to LCS, it is not the only acceptable approach for closing this care gap. Specifically, patients can be screened for lung cancer through other chest CTs (eg, diagnostic chest CTs) conducted for purposes other than LCS. Furthermore, patients may decline screening after SDM given that there are substantial potential downsides to screening and wide individual variation in patient lung cancer risk, life expectancy, and potential net benefit from screening.^11^ Therefore, other chest CTs or LCS SDM should be considered in addition to LDCT as valid approaches to LCS care gap closure.

This study’s primary goal was to provide a more holistic understanding of the association of a multifaceted, EHR-based CDS intervention with LCS care gap closure by accounting for these various approaches. A secondary goal was to evaluate whether providing simple patient portal reminders for LCS-eligible patients was associated with additional improvements in care cap closure. The primary study objective was to evaluate changes in LCS care gap closure via any means (ie, LDCT, other chest CT, or SDM) after the introduction of a multifaceted, EHR-integrated clinician-facing intervention (period 1) and the addition of patient-facing EHR patient portal reminders (period 2).

## Methods

### Design

This nonrandomized controlled trial followed a interrupted time series (ITS) design, encompassing a baseline period (usual care) lasting 12 months (August 24, 2019, to August 23, 2020), an intervention period 1 (clinician-facing intervention) lasting 11 months (August 24, 2020, to July 27, 2021), and an intervention period 2 (patient-facing intervention) lasting 9 months (July 28, 2021, to April 27, 2022). The study was approved by the University of Utah Institutional Review Board and registered with ClinicalTrials.gov (NCT04498052; see trial protocol in Supplement 1). There were no significant deviations from the registered trial. A waiver of consent was approved by the University of Utah Institutional Review Board because the intervention, which follows USPSTF guidelines, does not add more risk than the current standard of care and measures were in place to minimize privacy risks. Data were obtained from the UUH Enterprise Data Warehouse on September 14, 2023. This report follows the Transparent Reporting of Evaluations With Nonrandomized Designs (TREND) reporting guideline.

### Setting

The research was carried out across 28 primary care and 4 pulmonary clinics located at 12 UUH locations. UUH uses a decentralized approach to LCS whereby frontline clinicians, such as primary care clinicians and pulmonologists, refer eligible and interested patients for LCS. The UUH LCS program is accredited by the American College of Radiology, and the Huntsman Cancer Institute maintains a registry of patients who have undergone LCS. There were 2 clinics at 1 location that started the intervention but were excluded because they closed before study completion.

UUH uses the Epic EHR system (versions February 2019, August 2019, February 2020, August 2020, February 2021, and May 2022). The creation of the multifaceted LCS CDS intervention was led by a collaborative initiative known as ReImagine EHR,^12^ which applies interoperable EHR innovations to patient care.

### Participants

Study eligibility criteria were evaluated at the level of primary care visits (office visits or telehealth visits) during the study period. Patients were eligible if they met 2013 USPSTF LCS eligibility criteria at the time of the visit and had at least 1 primary care visit in the preceding year. Per USPSTF guidelines, a person qualified for LCS if they were aged 55 to 80 years, had a smoking history of 30 pack-years or greater, actively smoked or had quit in the previous 15 years, and had not been diagnosed with lung cancer.^2^ While USPSTF expanded these criteria in 2021,^13^ this analysis used 2013 criteria to maintain comparable patient populations across study periods. Inclusion and exclusion criteria were determined using EHR data on smoking, demographics, problem list entries, medical history, and encounter diagnoses.

### Interventions

**Figure 1.**
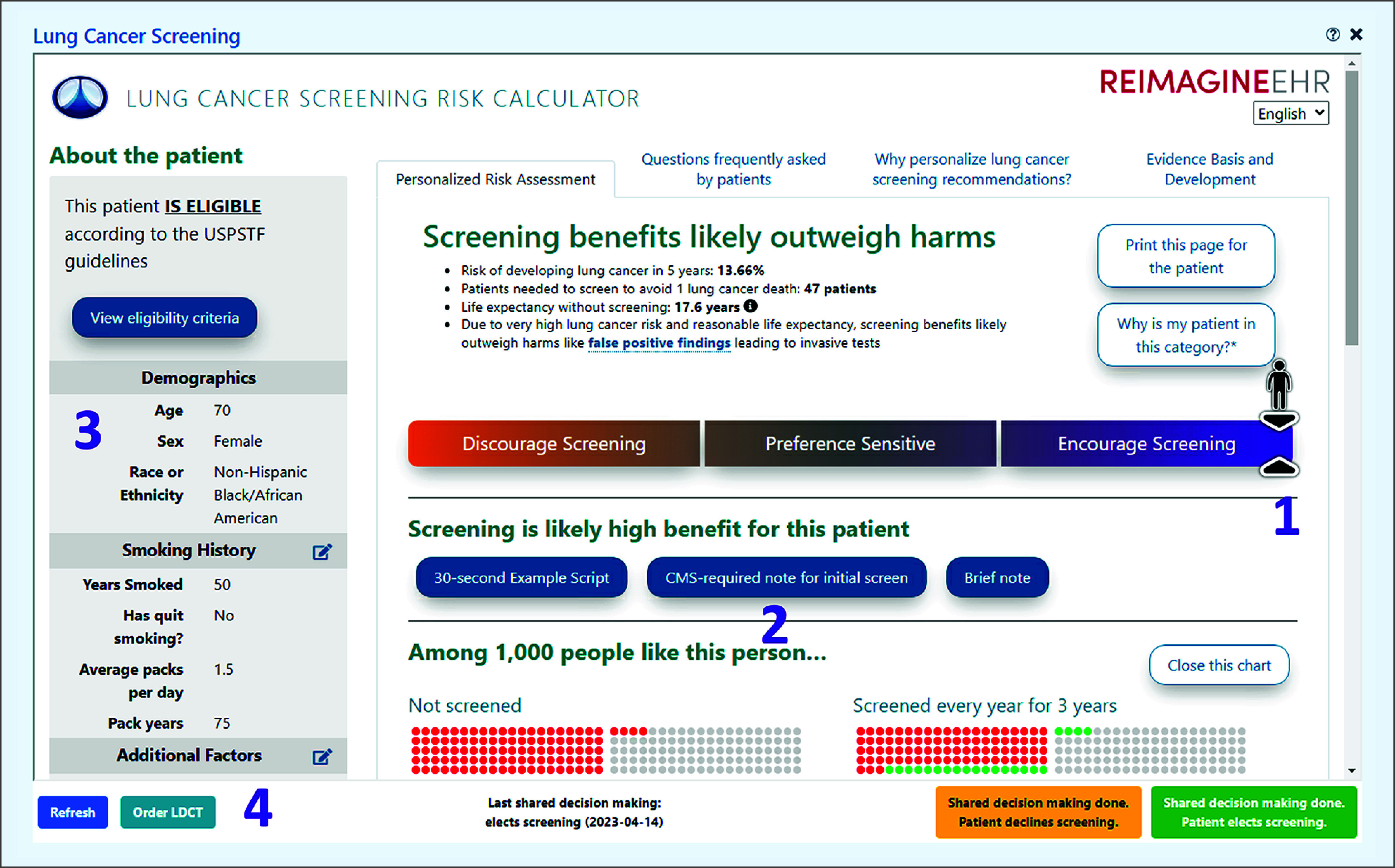
Shared Decision-Making App A screenshot of the electronic health record (EHR)–integrated lung cancer screening SDM tool is presented. Numbers 1 to 4 refer to key features: identification of patients expected to have a high benefit (1), note generation (2), input autopopulation (3), and 1-click ordering (4). LDCT indicates low-dose computed tomography; green circles, lives saved by screening; red circles, lung cancer deaths; USPSTF, US Preventive Services Task Force.

Period 1 CDS tools included clinician-facing reminders for LCS and LCS discussion in the EHR system Health Maintenance module, an EHR-integrated SDM tool (Figure 1), and narrative guidance provided in the LDCT ordering screen regarding LCS guidelines, including requirements from the Centers for Medicare & Medicaid Services to conduct SDM using a decision aid prior to initiating screening.^11^ The period 1 intervention was described previously^8^ and is detailed in the eMethods in Supplement 2.

In period 2, patient-facing reminders were added. These reminders were part of the EHR system Health Maintenance module, in which care gaps can be optionally presented to patients on the main portal screen. Reminders can also be accessed through the patient portal menu. Patient reminders consisted of notifications on the need for *Lung Cancer Screening* or *Lung Cancer Screening Discussion* based on whether patients required screening or an initial discussion on screening. No further information (eg, an explanation of LCS) was provided due to patient portal limitations.

In period 1, the SDM tool and clinician reminders supported 2013 USPSTF guidelines.^2^ Individualized predictions of net benefit in the SDM tool were based on the Bach risk model.^14,15^ In period 2, the SDM tool and clinician reminders were updated to support 2021 USPSTF guidelines.^13^ The SDM tool was updated to use the life-years gained from screening (LYFS)-CT model at this time to account for the higher risk of lung cancer among Black individuals; a threshold of at least 16.2 days of life expected to be gained from screening was used to identify patients expected to have a high benefit.^16^ Study interventions were communicated to users via typical channels for notifying clinicians regarding EHR system updates, including EHR-integrated prompts described previously and inclusion in brief updates on new EHR features presented at clinician meetings.

### Primary Outcomes

The primary outcome was LCS care gap closure through any means. Care gap closure through any means could be achieved through LDCT completion in the past year, completion of another chest CT in the past year, or SDM documentation in the past 3 years. To assess population care gap closure levels at the end of each study period, we estimated the care gap closure status for all patients who had primary care visits in the 12 months preceding the last day of the period. Using structured EHR data, we considered SDM documented if a clinician noted that the need for LCS discussion was addressed, the patient declined screening, or LCS was not appropriate.^8^ The primary hypothesis was that introduction of the multifaceted, EHR-integrated intervention would be associated with increased LCS care gap closure.

### Secondary Outcomes

Secondary outcomes included component mechanisms for achieving care gap closure (ie, through LDCTs, other chest CTs, and SDM), 3 mechanisms for unclosed care gaps (LCS ordered in past year but not completed, LCS elected in past 3 years but not ordered, and LCS not ordered and SDM not completed), and SDM tool use in the past 3 years. Of care gap closure mechanisms, closure through LDCTs was of particular interest.

### Covariates

To ensure that the patient population was stable across study periods, we assessed patient characteristics, including estimated screening benefit level,^16^ receipt of care in pulmonary clinics, sex, race and ethnicity, age, tobacco use, comorbidities, body mass index (calculated as weight in kilograms divided by height in meters squared), insurance status, family history of lung cancer, and patient portal use in the past year. The screening benefit level was reported in accordance with guidance from the American College of Chest Physicians to identify patients who might obtain the most benefit from screening (patients expected to have a high benefit).^17^ To identify these patients, we used LYFS-CT models, with a benchmark of an anticipated gain of at least 16.2 days of life.^16^ We identified 13 medical conditions or comorbidities from the problem list, medical history, and visit diagnoses. Race and ethnicity were derived from the EHR, where they were documented based on patient self-report as a part of routine clinical care. Race was documented as American Indian and Alaska Native, Asian, Black or African American, Native Hawaiian and Other Pacific Islander, White or Caucasian, and choose not to disclose. Ethnicity was documented as Hispanic or Latino, not Hispanic or Latino, and choose not to disclose. We aggregated race and ethnicity data into Hispanic or Latino, Black or African American, White or Caucasian, and other. The other race and ethnicity category included individuals of non-Hispanic ethnicity who selected a race of American Indian and Alaska Native, Asian, Native Hawaiian and Pacific Islander, or choose not to disclose. Non-Hispanic individuals were also categorized as other race and ethnicity if they selected more than 1 race or had no race selection in the system. Additionally, we reported Utah COVID-19 hospitalization rates as the 7-day mean of hospitalizations during the patient’s last eligible visit during each period.^18^ Patient characteristics were calculated as of the last date of each study period, with the last known observation carried forward for categorical variables. For overall patient demographics, we used the last date of the baseline period.

### Statistical Analysis

We used median and IQR to summarize continuous characteristics and count and percentage to summarize categorical characteristics. To compare characteristics across study periods, we used generalized linear models. To compare period 1 with baseline and period 2 with baseline, indicators of whether patients had visits in both periods were added to the model.

#### Primary Analysis: ITS

To evaluate the association of interventions with care gap closure rates, we conducted ITS analysis using the segmented regression approach. We chose segmented regression analysis because it allowed us to assess preexisting trends and changes in the slope and level of outcomes.^19^ Each patient was assigned a value of 1 (for care gap closure) or 0 (for care gap nonclosure) at the end of each month. The mean of these values for each month was found, and values were fitted into segmented linear regression models with study periods constituting 3 segments. We used the segmented least squares approach with parameters for intercept, baseline trend, and changes in the level and trend after the intervention. Care gap closure rates were stratified by estimated screening benefit level. We expected that implementing the tool would be associated with a higher rate of care gap closure in study period 1 compared with baseline, period 2 compared with period 1, and among patients with a high benefit compared with patients who were eligible but with intermediate benefit.

#### Secondary Analysis: Covariate Balancing Propensity Score

For primary and secondary outcome measures, we also estimated adjusted intervention outcomes after controlling for all characteristics using the covariate balancing propensity score (CBPS) approach.^20^ For binary outcomes, we used logistic regression. Improvements in covariate balance after propensity score adjustment are summarized in eFigure 2 in Supplement 2.

Statistical significance was defined at alpha = .05. We used R statistical software version 4.3.2 (R Project for Statistical Computing) and tidyverse, WeightIt, nnet, and betareg extension packages. Data were analyzed from September 2023 through February 2024.

## Results

### Patient Characteristics

There were 19 008 patients who met inclusion criteria (eTable 1 in Supplement 2). Using exclusion criteria consecutively, we excluded 7698 patients (40.5%) due to insufficient detailed smoking data in the EHR to determine eligibility, 10 847 patients (57.1%) due to not meeting USPSTF criteria for lung cancer screening based on detailed smoking data, and 75 patients (0.4%) due to lung cancer diagnosis. The study included 1865 patients (median [IQR] age, 64 [60-70] years; 759 female [40.7%] and 1106 male [59.3%]; 98 Hispanic [5.3%], 36 non-Hispanic Black or African American [1.9%], 1574 non-Hispanic White [84.4%], and 157 other race or ethnicity [8.4%]) who met eligibility criteria (9.8%) (eFigure 1, eTable 1, and eTable 2 in Supplement 2). A substantial proportion of patients had insufficient EHR smoking data to determine eligibility. Populations were similar in the 3 periods (eg, the median [IQR] age was 65.0 [60.8-71.0] at baseline, 65.0 [60.0-70.0] years in period 1, and 65.0 [60.0-70.0] years in period 2) (Table 1). The COVID-19 pandemic started during period 1. The pandemic was associated with increased use of the patient portal and telehealth visits.

**Table 1.** Patient Characteristics

Characteristic	Patients, No. (%)		*P* value^a^	Patients in intervention period 2, No. (%) (n = 1255)	*P* value^a^
	Baseline period (n = 1104)	Intervention period 1 (n = 1219)			
No. visits per patient, median (IQR)	3.0 (1.0-5.0)	3.0 (1.0-4.0)	NA	2.0 (1.0-4.0)	NA
High-benefit personalized screening benefit level	694 (62.9)	781 (64.1)	.56	795 (63.3)	.78
≥1 Pulmonary visit in study period	42 (3.8)	60 (4.9)	.20	49 (3.9)	.97
Sex assigned at birth					
Female	470 (42.6)	486 (39.9)	.24	514 (41.0)	.47
Male	634 (57.4)	733 (60.1)		714 (59.0)	
Race and ethnicity					
Black or African American, non-Hispanic	20 (1.8)	21 (1.7)	.98	25 (2.0)	.54
Hispanic, any race	59 (5.3)	63 (5.2)		66 (5.3)	
White, non-Hispanic	946 (85.7)	1042 (85.5)		1053 (83.9)	
Other^b^	79 (7.2)	93 (7.6)		111 (8.8)	
Age, median (IQR), y	65.0 (60.8-71.0)	65.0 (60.0-70.0)	.91	65.0 (60.0-70.0)	.50
Tobacco use					
Time smoked, median (IQR), y	40.0 (30.0-45.0)	40.0 (30.0-45.0)	.11	40.0 (30.0-45.6)	.04
Cigarettes per day, median (IQR)	20.0 (20.0-30.0)	20.0 (20.0-30.0)	.59	20.0 (20.0-30.0)	.03
Smoking history					
Current smoker	550 (49.8)	629 (51.6)	.58	645 (51.4)	.53
Former smoker (quit <15 y ago)	554 (50.2)	590 (48.4)		610 (48.6)	
Time since last smoked, median (IQR), y	7.0 (3.9-10.7)	7.3 (3.4-11.1)	.98	6.9 (3.3-10.4)	.20
Insurance					
Commercial	337 (30.5)	370 (30.4)	.11	380 (30.3)	.06
Government	725 (65.7)	820 (67.3)		847 (67.5)	
Self-pay	42 (3.8)	29 (2.4)		28 (2.2)	
Patient portal use in preceding year	700 (63.4)	881 (72.3)	<.001	963 (76.7)	<.001
Telehealth primary care visit in preceding year	398 (36.1)	511 (41.9)	<.001	470 (37.5)	.20
COVID-19 hospitalizations per day in Utah, median (IQR)	15.9 (0.0-25.9)	25.1 (20.6-34.7)	<.001	37.3 (20.1-62.0)	<.001

Abbreviation: NA, not applicable.

^a^ *P* values are based on generalized linear models and are all vs baseline.

^b^ The other race and ethnicity group consisted of individuals of non-Hispanic ethnicity with the following race responses: American Indian and Alaska Native, Asian, Native Hawaiian and Pacific Islander, more than 1 race, and chose not to disclose.

### Primary Analysis: ITS

**Figure 2.**
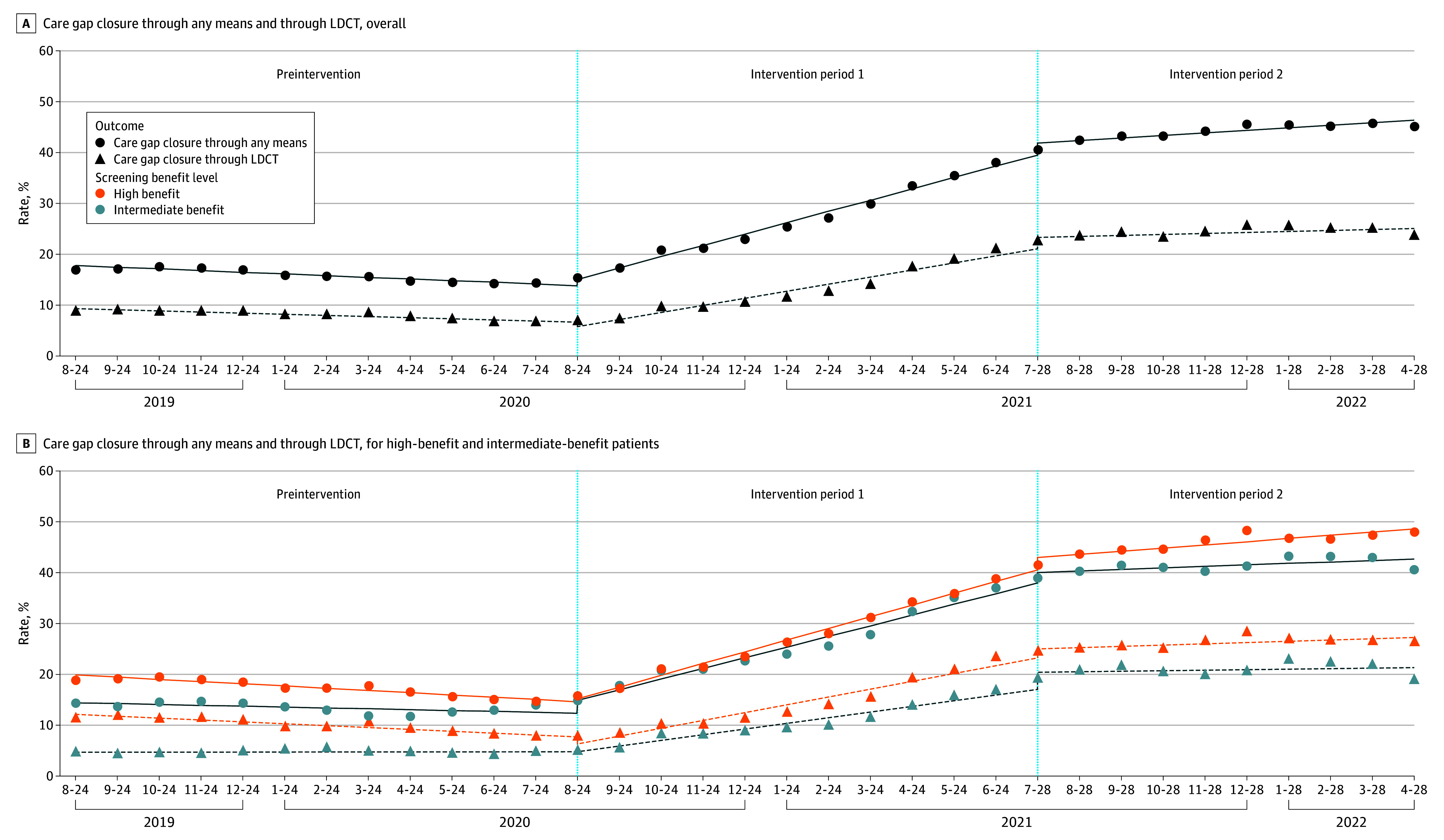
Changes in Primary Outcomes Lung cancer screening care gap closure through any means and low-dose computed tomography (LDCT)–based gap closure is presented overall (A) and by patient benefit level (B).

In the baseline period, the LCS care gap closure through any means and LCS care gap closure through LDCT were 17.8% (95% CI, 17.0% to 18.6%) and 9.2% (95% CI, 19.0% to 20.8%), respectively, and their slopes were −0.3% (95% CI, −0.5% to −0.2%) per month and −0.2% (95% CI, −0.4% to −0.1%) per month, respectively (Table 2 and Figure 2). In intervention period 1 (clinician-facing intervention), changes in care gap closure and LDCT-based closure levels were not significant, but changes in their slopes were significant, at 2.6 percentage points (95% CI (95% CI, 2.4 to 2.7 percentage points) per month and 1.6 percentage points (95% CI, 1.4 to 1.8 percentage points) per month, respectively. In intervention period 2 (clinician- and patient-facing reminders), care gap closure and LDCT-based closure levels increased by 2.3 percentage points (95% CI, (1.0 to 3.6 percentage points) and 2.4 percentage points (95% CI, 0.9 to 3.9 percentage points), respectively, and their slopes decreased by −1.7 percentage points (95% CI, −1.9 to −1.5 percentage points) per month and −1.2 percentage points (95% CI, −1.4 to −0.9 percentage points) per month, respectively. Analyses stratified by patient benefit level showed similar trends as the overall analysis. The increase in the slope of LDCT completion in period 1 was higher for patients with a high benefit compared with those with an intermediate benefit (1.9 percentage points; 95% CI, 1.6 to 2.1 percentage points per month vs 1.1 percentage points; 95% CI, 0.9 to 1.4 percentage points per month).

**Table 2.** ITS Analysis: Association of Intervention With Care Gap Closure

Means of care gap closure	Before intervention			Intervention period 1				Intervention period 2			
	Level (95% CI), %	Slope (95% CI), %/mo	*P* value	Level change (95% CI), percentage points	*P* value	Slope change (95% CI), percentage points/mo	*P* value	Level change (95% CI), percentage points	*P* value	Slope change (95% CI), percentage points/mo	*P* value
Any											
Overall	17.8 (17.0 to 18.6)	−0.3 (−0.5 to −0.2)	<.001	1.2 (0.0 to 2.5)	.05	2.6 (2.4 to 2.7)	<.001	2.3 (1.0 to 3.6)	.001	−1.7 (−1.9 to −1.5)	<.001
Patients with high benefit	19.9 (19.0 to 20.8)	−0.4 (−0.6 to −0.3)	<.001	0.6 (−0.8 to 2.0)	.41	2.7 (2.5 to 3.0)	<.001	2.4 (0.9 to 3.9)	.002	−1.7 (−1.9 to −1.4)	<.001
Patients with intermediate benefit	14.4 (13.1 to 15.6)	−0.2 (−0.4 to 0.0)	.08	2.5 (0.6 to 4.4)	.01	2.3 (2.0 to 2.6)	<.001	2.1 (0.1 to 4.1)	.04	−1.8 (−2.2 to −1.5)	<.001
LDCT											
Overall	9.2 (8.3 to 10.1)	−0.2 (−0.4 to −0.1)	.005	−1.0 (−2.4 to 0.4)	.17	1.6 (1.4 to 1.8)	<.001	2.4 (0.9 to 3.9)	.002	−1.2 (−1.4 to −0.9)	<.001
Patients with high benefit	12.1 (11.0 to 13.1)	−0.4 (−0.5 to −0.2)	<.001	−1.4 (−3.0 to 0.3)	.09	1.9 (1.6 to 2.1)	<.001	1.8 (0.1 to 3.5)	.04	−1.3 (−1.6 to −1.0)	<.001
Patients with intermediate benefit	4.6 (3.6 to 5.7)	0.0 (−0.2 to 0.2)	.90	−0.1 (−1.7 to 1.5)	.88	1.1 (0.9 to 1.4)	<.001	3.4 (1.7 to 5.1)	<.001	−1.0 (−1.3 to −0.7)	<.001

Abbreviations: ITS, interrupted time series; LDCT, low-dose computed tomography.

### Secondary Analysis: CBPS

Regression analysis results are summarized in Table 3 with adjusted ORs estimated via CBPS. This analysis confirmed results for the ITS analysis. The intervention was associated with an increase in LCS care gap closure, from 175 of 1104 patients (15.9%) at the end of the baseline period to 510 of 1219 patients (41.8%) at the end of intervention period 1 (adjusted OR vs baseline, 3.7; 95% CI, 3.04.6) and 588 of 1255 patients (46.9%) at the end of intervention period 2 (adjusted OR vs baseline, 4.3; 95% CI, 3.5,-5.3). At the end of intervention period 2, most patients (298 patients [23.7%]) achieved care gap closure though LDCT, followed by documentation of SDM in Health Maintenance modules (159 patients [12.7%]) and other chest CT (131 patients [10.4%]). For 107 patients in period 2 [8.5%], LCS was ordered but not completed. The number of patients for whom neither SDM nor LCS were completed decreased from 889 patients (80.5%) at baseline to 547 patients (43.6%) at the end of intervention period 2 (adjusted OR vs baseline, 0.2; 95% CI, 0.2-0.2). The SDM tool was used for 168 of 1255 eligible patients (13.4%) in period 2.

**Table 3.** CBPS Analysis: Association of Intervention With Study Outcomes

Outcome	Patients, No. (%)		aOR (95% CI)	*P* value^b^	Patients in intervention period 2, No. (%) (n = 1255)^a^	aOR (95% CI)^c^	*P* value^b^
	Baseline (n = 1104)^a^	Intervention period 1 (n = 1219)^a^					
LCS care gap closure rate							
Overall	175 (15.9)	510 (41.8)	3.7 (3.0-4.6)	<.001	588 (46.9)	4.3 (3.5-5.3)	<.001
Through LDCT	75 (6.8)	278 (22.8)	3.6 (2.7-4.7)	<.001	298 (23.7)	3.5 (2.6-4.6)	<.001
Through other chest CT	93 (8.4)	126 (10.3)	1.2 (0.9-1.6).	.31	131 (10.4)	1.4 (1.0-1.9)	.03
Through SDM	7 (0.6)	106 (8.7)	25.3 (9.8-65.8)	<.001	159 (12.7)	29.0 (11.3-74.3)	<.001
LCS nonclosure rate							
Overall	929 (84.1)	709 (58.2)	0.3 (0.2-0.3)	<.001	667 (53.1)	0.2 (0.2-0.3)	<.001
LCS ordered in past year but not completed	39 (3.5)	88 (7.2)	1.8 (1.2-2.7)	.002	107 (8.5)	2.4 (1.6-3.5)	<.001
LCS elected in past 3 y but not ordered	1 (0.1)	2 (0.2)	NA	NA	13 (1.0)	NA	NA
LCS not ordered and SDM not completed	889 (80.5)	619 (50.8)	0.3 (0.2-0.3)	<.001	547 (43.6)	0.2 (0.2-0.2)	<.001
SDM tool use in past 3 y or on visit date	NA	140 (11.5)	NA	NA	168 (13.4)	NA	NA

Abbreviations: aOR, adjusted odds ratio; CT, computed tomography; LCS, lung cancer screening; LDCT, low-dose computed tomography; NA, not applicable; SDM, shared decision-making.

^a^ Estimates are at the end of each period.

^b^ All comparisons are vs the baseline period.

^c^ Adjusted ORs were calculated based on the propensity score approach.

Most of the increase in care-gap closure was contributed by increases in LDCT and LCS SDM. Care gap closure through LDCT increased from 75 patients (6.8%) in the baseline period to 278 patients (22.8%) in period 1 (adjusted OR vs baseline, 3.6; 95% CI, 2.7-4.7) and 298 patients (23.7%) in period 2 (adjusted OR vs baseline, 3.5; 95% CI, 2.6-4.6), while care gap closure through LCS SDM increased from 7 patients (0.6%) in the baseline period to 106 patients (8.7%) in period 1 (adjusted OR vs baseline, 25.3; 95% CI, 9.8-65.8) and 159 patients (12.7%) in period 2 (adjusted OR vs baseline, 29.0; 95% CI, 11.3-74.3) and care-gap closure through other chest CT increased from 93 patients (8.4%) in the baseline period to 126 patients (10.3%) in period 1 (adjusted OR vs baseline, 1.2; 95% CI, 0.9-1.6) and 131 patients (10.4%) in period 2 (adjusted OR vs baseline, 1.4; 95% CI, 1.0-1.9).

## Discussion

This nonrandomized controlled trial found that introduction of a multifaceted, EHR-integrated intervention was associated with improved LCS care gap closure in an academic health care system. Introduction of clinician-facing interventions was associated with improvement (period 1), with a slight further increase associated with the addition of patient-facing reminders (period 2). Although the care gap closure rate slowed in period 2, eligible patients continued to have their care gaps closed throughout the study. While both clinician- and patient-facing LCS interventions showed potential benefits, 43.6% of screening-eligible patients still lacked LDCT orders or discussions at the end of period 2. These findings suggest that further implementation strategies are needed to improve LCS care gap closure.

Study analyses considered care gap closure as a multifactor outcome, including LDCT completion, other chest CTs, and SDM documentation. These alternative approaches to care gap closure accounted for a substantial proportion of overall closures, with 10.4% attributed to other chest CTs and 12.7% to SDM documentation, compared with 23.7% for LDCT. Notably, the National Committee for Quality Assurance announced plans to introduce a Healthcare Effectiveness Data and Information Set quality measure for LCS; however, it is unclear whether these measures would address alternative strategies to care gap closure.^21^ While such quality measures are defined, findings of our study suggest the need to account for LCS care gap closures through not only LDCT, but also other chest CTs and SDM.

This study has several strengths. It evaluated a multifaceted, standards-based, and EHR-integrated intervention that could potentially be widely scaled.^8^ Furthermore, this study evaluated additive outcomes associated with simple patient portal reminders using detailed smoking history in the EHR to assess eligibility; this has not been studied to date, to our knowledge. Additionally, this study found that other chest CTs and SDM combined were approximately as common (23.1%) as LDCTs (23.7%) for closing LCS care gaps in phase 2. This is consistent with prior literature identifying that most patients have lung cancer identified through chest imaging outside of LDCTs.^22^

This study identified several areas of need for further research and improvement. As identified by us and others previously^23,24,25,26^ and as underscored by the large number of patients with unknown LCS eligibility in this study, there is a need to improve the documentation of detailed smoking history in the EHR. Moreover, 8.5% of patients in this study had an LDCT ordered in the past year but did not complete it, indicating the need for improving follow-through after LDCT ordering. Even with clinician- and patient-facing interventions, LCS care gaps remained in more than half of LCS-eligible patients, and the LCS tool was used with only 13.4% of eligible patients, suggesting the need for further research to test implementation strategies to improve SDM and LCS. Additionally, as shown by the modest change in outcome associated with the simple patient portal reminder, more research may be needed on improving patient education and empowerment through more engaging patient-facing interventions. Accordingly, a new multisite trial is underway to evaluate the additive outcome associated with offering more robust patient-facing interventions, including individualized patient education through the patient portal.

### Limitations

This study has several limitations. First, we used a nonrandomized ITS study design without parallel controls. The onset of the COVID-19 pandemic during the baseline period posed potential biases, such as reduced LCS rates due to diminished clinician capacity. We believe that the small increase in other chest CTs may be attributed to the COVID-19 pandemic rather than to our intervention. Furthermore, USPSTF guideline changes in 2021 resulted in a larger number of patients becoming eligible for LCS. We used more stringent 2013 USPSTF criteria to maintain compatibility with period 1, but we were unable to fully separate the intervention outcome from potential outcomes associated with the guideline change itself. To mitigate the impact of this limitation, we used propensity score analysis techniques to account for differences in covariates. Second, our study took place in a single academic health care system, limiting generalizability. Third, we depended on EHR smoking history data, which may underestimate patient eligibility due to inaccuracies.^23^ Fourth, the quality of SDM was not evaluated. Fifth, due to the limited quality of EHR smoking data, a large proportion of individuals had insufficient detailed smoking history data to determine their eligibility. We are currently implementing follow-up interventions aimed at improving the identification of patients eligible for LCS.

## Conclusions

In this nonrandomized controlled trial, implementing a comprehensive intervention that combined clinician-facing EHR reminders, an EHR-integrated SDM tool for personalized screening, narrative guidance presented in the LDCT order screen, and patient-facing reminders was associated with an increase in LCS care gap closure. Further research is needed to improve LCS, including through improved documentation of detailed smoking history in the EHR, improved LDCT follow-through rates, and more effective engagement of patients in their care.

## Notes

Supplement 1.
Trial Protocol and Statistical Analysis Plan

Supplement 2. **eMethods.** Details of Clinician-Facing Period 1 Intervention **eFigure 1.** Study Flowchart **eTable 1.** Patient Inclusion and Exclusion Criteria **eTable 2.** Overall Patient Population Demographics **eFigure 2.** Covariate Balancing Propensity Score Model and Improvement in Covariates Balance for Baseline and Intervention Periods 1 and 2

Supplement 3.
Data Sharing Statement

## References

[1] Siegel RL, Miller KD, Wagle NS, Jemal A. Cancer statistics, 2023. CA Cancer J Clin. 2023;73(1):17-48. doi:10.3322/caac.21763 36633525

[2] US Preventive Services Task Force. Final recommendation statement: lung cancer screening. Accessed June 1, 2021. https://www.uspreventiveservicestaskforce.org/uspstf/recommendation/lung-cancer-screening-december-2013

[3] Krist AH, Davidson KW, Mangione CM, ; US Preventive Services Task Force. Screening for lung cancer: US Preventive Services Task Force recommendation statement. JAMA. 2021;325(10):962-970. doi:10.1001/jama.2021.1117 33687470

[4] de Koning HJ, van der Aalst CM, de Jong PA, . Reduced lung-cancer mortality with volume CT Screening in a randomized trial. N Engl J Med. 2020;382(6):503-513. doi:10.1056/NEJMoa1911793 31995683

[5] Aberle DR, Adams AM, Berg CD, ; National Lung Screening Trial Research Team. Reduced lung-cancer mortality with low-dose computed tomographic screening. N Engl J Med. 2011;365(5):395-409. doi:10.1056/NEJMoa1102873 21714641 PMC4356534

[6] Fedewa SA, Bandi P, Smith RA, Silvestri GA, Jemal A. Lung cancer screening rates during the COVID-19 pandemic. Chest. 2022;161(2):586-589. doi:10.1016/j.chest.2021.07.030 34298006 PMC8294072

[7] Reese TJ, Schlechter CR, Kramer H, . Implementing lung cancer screening in primary care: needs assessment and implementation strategy design. Transl Behav Med. 2022;12(2):187-197. doi:10.1093/tbm/ibab115 34424342 PMC8848991

[8] Kukhareva PV, Li H, Caverly TJ, . Implementation of lung cancer screening in primary care and pulmonary clinics: pragmatic clinical trial of electronic health record-integrated everyday shared decision-making tool and clinician-facing prompts. Chest. 2023;164(5):1325-1338. doi:10.1016/j.chest.2023.04.040 37142092 PMC10792294

[9] Steinberg MB, Young WJ, Miller Lo EJ, . Electronic health record prompt to improve lung cancer screening in primary care. Am J Prev Med. 2023;65(5):892-895. doi:10.1016/j.amepre.2023.05.016 37306638

[10] Liu S, McCoy AB, Aldrich MC, . Leveraging natural language processing to identify eligible lung cancer screening patients with the electronic health record. Int J Med Inform. 2023;177:105136. doi:10.1016/j.ijmedinf.2023.105136 37392712 PMC11537206

[11] Centers for Medicare & Medicaid Services. Decision memo: screening for lung cancer with low dose computed tomography (LDCT): CAG-00439R). Accessed April 30, 2024. https://www.cms.gov/medicare-coverage-database/view/ncacal-decision-memo.aspx?proposed=N&ncaid=304

[12] Kawamoto K, Kukhareva PV, Weir C, . Establishing a multidisciplinary initiative for interoperable electronic health record innovations at an academic medical center. JAMIA Open. 2021;4(3):ooab041. doi:10.1093/jamiaopen/ooab041 34345802 PMC8325485

[13] US Preventive Services Task Force. Final recommendation statement: lung cancer screening. Accessed June 1, 2021. https://www.uspreventiveservicestaskforce.org/uspstf/recommendation/lung-cancer-screening

[14] Bach PB, Elkin EB, Pastorino U, . Benchmarking lung cancer mortality rates in current and former smokers. Chest. 2004;126(6):1742-1749. doi:10.1378/chest.126.6.1742 15596668

[15] Bach PB, Kattan MW, Thornquist MD, . Variations in lung cancer risk among smokers. J Natl Cancer Inst. 2003;95(6):470-478. doi:10.1093/jnci/95.6.470 12644540

[16] Cheung LC, Berg CD, Castle PE, Katki HA, Chaturvedi AK. Life-gained-based versus risk-based selection of smokers for lung cancer screening. Ann Intern Med. 2019;171(9):623-632. doi:10.7326/M19-1263 31634914 PMC7191755

[17] Mazzone PJ, Silvestri GA, Souter LH, . Screening for lung cancer: CHEST guideline and expert panel report. Chest. 2021;160(5):e427-e494. doi:10.1016/j.chest.2021.06.063 34270968 PMC8727886

[18] Utah Department of Health and Human Services. Respiratory disease surveillance. Updated April 24, 2024. Accessed July 17, 2022. https://coronavirus.utah.gov/case-counts/

[19] Penfold RB, Zhang F. Use of interrupted time series analysis in evaluating health care quality improvements. Acad Pediatr. 2013;13(6)(suppl):S38-S44. doi:10.1016/j.acap.2013.08.002 24268083

[20] Imai K, Ratkovic M. Covariate balancing propensity score. J R Stat Soc Series B Stat Methodol. 2014;76(1):243-263. doi:10.1111/rssb.12027

[21] Reynolds A. New measure coming for lung cancer screening. National Committee for Quality Assurance. Accessed September 13, 2023. https://www.ncqa.org/blog/new-measure-coming-for-lung-cancer-screening/

[22] Gieske MR, Kerns J, Schmitt GM, . Overcoming barriers to lung cancer screening using a systemwide approach with additional focus on the non-screened. J Med Screen. Published online October 19, 2023. doi:10.1177/09691413231208160 37855047

[23] Kukhareva PV, Caverly TJ, Li H, . Inaccuracies in electronic health records smoking data and a potential approach to address resulting underestimation in determining lung cancer screening eligibility. J Am Med Inform Assoc. 2022;29(5):779-788. doi:10.1093/jamia/ocac020 35167675 PMC9006678

[24] Cole AM, Pflugeisen B, Schwartz MR, Miller SC. Cross sectional study to assess the accuracy of electronic health record data to identify patients in need of lung cancer screening. BMC Res Notes. 2018;11(1):14. doi:10.1186/s13104-018-3124-0 29321038 PMC5763525

[25] Modin HE, Fathi JT, Gilbert CR, . Pack-year cigarette smoking history for determination of lung cancer screening eligibility: comparison of the electronic medical record versus a shared decision-making conversation. Ann Am Thorac Soc. 2017;14(8):1320-1325. doi:10.1513/AnnalsATS.201612-984OC 28406708

[26] Polubriaginof F, Salmasian H, Albert DA, Vawdrey DK. Challenges with collecting smoking status in electronic health records. AMIA Annu Symp Proc. 2018;2017:1392-1400. 29854208 PMC5977725

