# Supplementary material for: Lung Cancer Screening Before and After a Multifaceted Electronic Health Record Intervention: A Nonrandomized Controlled Trial
Source: JAMA Netw Open. 2024 Jun 7;7(6):e2415383. doi: 10.1001/jamanetworkopen.2024.15383 (PMC11161845; doi:10.1001/jamanetworkopen.2024.15383)
Supplement: Supplement 1. — Trial Protocol and Statistical Analysis Plan [file jamanetwopen-e2415383-s001.pdf]

Design and Implementation of Scalable Decision Support and Shared Decision Making for Lung Cancer Screening

Protocol Summary

**IRB Approval Date of Current Version:**	3/14/2023
**University of Utah IRB #:**	IRB_00125797
**Sponsor:**	DHHS AGNCY FOR HEALTHCARE RESH & QUALITY
**Principal Investigator:**	Kensaku Kawamoto
**Internal Staff and Sub-Investigators:**	**Site Name**: University of Utah; **Staff Names**: [redacted]

This document was created using the ERICA Online System at the University of Utah. The document is created from study information approved by the IRB on the date listed above. Any alteration to the original content of this document may not be considered to represent the study as approved by the IRB.

Background and Introduction

Lung cancer is prevalent and deadly. Lung cancer is the second most commonly diagnosed cancer in the United States (US), with an estimated 234,030 new diagnoses in 2018, and it is the leading cause of cancer-related deaths among both men and women, with an estimated 150,050 deaths in 2018.[1] Lung cancer accounts for approximately 1 in 4 cancer-related deaths.[1] The primary risk factor for lung cancer is cigarette smoking.[1]

Low-dose computed tomography (LDCT) screening is effective for reducing lung cancer mortality. The National Lung Screening Trial (NLST)[2] enrolled 53,454 participants aged 55 to 74 years who had a history of cigarette smoking of at least 30 pack-years and, if former smokers, had quit within the past 15 years. This study randomized participants to screening with either LDCT or chest radiography. Compared to the radiography group, LDCT resulted in a 20% relative reduction in lung cancer mortality (p = 0.004).[2] This 20% relative reduction in lung cancer deaths with screening is larger than the reduction in breast cancer deaths with mammograms. Based on this finding,[2] the US Preventive Services Task Force (USPSTF) recommended in 2013 that annual LDCT be performed for lung cancer screening in patients meeting NLST enrollment criteria.[3,4] The USPSTF recommendations expand the eligible age range from 55 to 80 years inclusive. The US Preventive Services Task Force (USPSTF) provided a grade B recommendation in 2013 that annual LDCT lung cancer screening be offered to patients 55 to 80 years old with a 30+ pack-year smoking history who are current smokers or quit in the last 15 years.[3,4] In March 2021, the USPSTF updated its recommendation to lower the eligibility age to 50 and the smoking requirement to 20+ pack-years.[5] The revised criteria should reduce lung cancer mortality by an additional 33% while increasing the number of screening-eligible patients by 67%.[6]

LDCT can result in significant harm, with the balance of benefits and harms dependent on the patient's risk profile. The NLST found substantial harms associated with LDCT screening, including high false positive rates (96.4%) which can lead to unnecessary and potentially harmful invasive procedures.[2] Moreover, additional studies found that the balance of benefits and harms is highly dependent on the patient's risk profile.[7–9] These studies developed validated models for predicting the likelihood of lung cancer, false positive LDCT tests, and adverse events based on patient-specific risk factors including age, gender, smoking history, and asbestos exposure.[7–9] Among LDCT-eligible patients, the ratio of false positive results to LDCT-prevented lung cancer death range from 1,648 among those in the lowest quintile of risk to 65 for those in the highest quintile of risk.[8]

Shared decision making (SDM) ensures that patients and providers make informed, patient-centered decisions regarding LDCT screening. Because the net benefit of LDCT screening can vary dramatically across eligible patients, the USPSTF strongly advocates for SDM, asserting that "the decision to begin screening should be the result of a thorough discussion of the possible benefits, limitations, and known and uncertain harms."[3] In a 2015 decision memo, the Centers for Medicare and Medicaid Services (CMS) required a SDM consultation involving use of a decision aid for payment of initial LDCT screening.[10] SDM is the process wherein the

provider shares medical information about options and patients share their values and preferences. Following the sharing of information by both parties, the patient and provider collaborates to decide on the course of action that reflects both the best medical evidence and the patient's preferences and values.[11,12]

Current LDCT screening rates among eligible patients is <5% nationally. Despite the USPSTF recommending LDCT in 2013, the percentage of eligible smokers who received LDCT screening in the past 12 months remained low and constant, from 3.3% in 2010 to 3.9% in 2015,[13] and a more recent analysis found no improvements.[14] At University of Utah Health, 6.4% of eligible patients received LDCT screening in year preceding August 2018.

The benefits of LDCT screening outweigh even highly negative patient views ~50% of the time. In a recent study led by co-I Caverly, a state-transition microsimulation model was developed to evaluate the impact of both patient risk and patient preferences.[15] This study found that for ~50% of the study population at the highest risk, the benefits of LDCT screening overcame even highly negative views about screening and its downsides.

If eligible patients in the top 60% of risk were screened, ~10,000 lung cancer deaths could be averted each year in the US. This represents 88% of the possible lung cancer mortality benefits from screening.[8] Since screening of all USPSTF-eligible patients could avert 12,250 lung cancer deaths per year in the US,[16] and given the existing ~5% screening rate, appropriate SDM and LDCT could avert up to ~10,000 lung cancer deaths per year if patients in the top 60% of risk were screened (12,500 if all screened * 0.88 benefit from screening top 60% * 0.95 not yet screened = 10,450). As noted earlier, the benefits of LDCT screening should outweigh any negative patient preferences for ~50% of patients. Also, of 4,246 LDCT-eligible patients who were offered screening and paper-based SDM at eight medical centers in the Veterans Health Administration (VHA), 58% agreed to undergo screening and 50% underwent screening during the study timeframe.[17] Thus, we believe a screening rate of 40-60% is a reasonable expectation if all eligible patients underwent effective SDM for LDCT. Even if only the patients at the top 40% of risk were screened, 73% of the lung cancer mortality benefits from screening would be achieved, translating to ~8,700 lung cancer deaths averted annually in the US.[8]

The level of evidence underlying these recommendations is substantial. Through its systematic evidence review, the USPSTF concluded that LDCT screening should be offered to eligible patients due to moderate certainty of substantial net benefit (evidence grade B).[3] Moreover, the mandate by CMS and the USPSTF that SDM be performed prior to LDCT screening reflects the strong evidence that SDM improves outcomes. Research has found that patients who share in medical decision making tend to be more satisfied with their care,[18] report a better quality of life,[19] and are more adherent to recommendations than those who do not.[20]

Stand-alone, Web-based CDS tools are available for LDCT SDM, including a tool developed by the project team known as Decision Precision that uses a continuous risk model. In recognition of the need to support SDM for LDCT screening, several groups, including

AHRQ, have developed stand-alone, Web-based CDS tools in this area.[21–24] An important limitation of these other tools is that they use trial averages rather than provide personalized guidance based on a continuous model. Our project leverages a provider-facing, Web-based CDS and SDM tool known as "Decision Precision" that uses a continuous model for providing patient-specific guidance. Decision Precision supports the USPSTF guidelines for LDCT screening[5] while providing patient-specific information on the expected benefits and harms of screening.[7–9,25] This Web tool provides (1) personalized quantitative risk assessment of the trade-offs; (2) patient-friendly language; (3) graphics that have been empirically demonstrated to help patients understand their personalized benefits and harms; and (4) quick and easy documentation of personalized SDM after using the tool. The system also supports printing a patient handout that includes patient-specific benefit and harm information, whether he/she is eligible for LDCT screening per USPSTF guidelines, guidance on smoking cessation, information on LDCT, and what happens next if the screening test is positive. Decision Precision also generates a summary of the SDM session that can be copied into the EHR for documentation.

Decision Precision has been iteratively enhanced through usability studies and pilot testing. To determine the best risk communication method for presenting the benefits and harms of lung cancer screening, we first conducted a randomized survey experiment with 1,612 adult smokers. We compared comprehension and perception of lung cancer screening benefits and harms when information was presented using four different evidence-based formats. Participants who viewed a pictograph had better knowledge about the magnitude of the benefit and how this benefit compared with important harms, leading to our use of pictographs in the tool. In addition, we iteratively designed four versions of the Web tool based on usability testing with decision aid researchers, primary care providers (PCPs), and patients. Decision Precision has been further refined through feedback from providers and patients who have helped further develop the tool at 4 VHA medical centers: Ann Arbor, Durham, Portland, and Charleston. The tool has been used to help guide discussions about lung cancer screening with >1,100 patients. Table 1 outlines key lessons learned and how they will be applied in this project.

Table 1. Key Lessons Learned from Clinical Use of Decision Precision and Incorporation into Project

Key Lesson	Incorporation into Project
Clinicians are used to binary decisions based on yes/no cut-offs rather than SDM weighing continuous risks and benefits.	Decision Precision now specifies when screening is of high benefit and likely to overcome even highly negative patient preferences.[15]
Incorporating time-consuming SDM can be challenging in the context of busy and time-constrained clinic visits.	Explore leveraging non-physician care team members for SDM. Enable patients to review Decision Precision+ results at home between visits.
User training is important, but must be time-efficient. One site declined to	User training for Decision Precision+ has been made as efficient as possible, so that it can be deployed in contexts without external funding.

participate, citing workload and time demands.	
Local workflows can vary greatly. A scalable CDS solution must not only account for variations in health IT platforms across health systems but also variations in workflow.	Leverage extensive workflow assessments, including a PCORI-funded assessment of smoking-related clinical workflows being conducted at over 30 community primary care practices across the state of Utah.
Response rates to paper-based patient surveys are decent (27%) but could be improved.	Patient surveys will both be mailed and administered electronically using the patient questionnaire feature of the personal health record (PHR).

Stand-alone CDS tools are limited by a lack of workflow integration and duplicate data entry. Stand-alone, Web-based CDS tools such as Decision Precision are easy to deploy. However, they have significant limitations: (1) they are not integrated into EHRs and routine workflows; (2) they require manual, often duplicative data entry; and (3) patients and providers are often unaware of their existence. In the context of busy clinical practices, stand-alone, Web-based CDS tools have a significant risk of limited impact due to suboptimal use.[26,27]

Integration of CDS with the EHR can overcome limitations of stand-alone tools, but widely scaling such CDS tools is difficult. Integrated CDS tools, as opposed to stand-alone CDS tools, (1) integrate the CDS with usual clinical workflows and (2) remove the need for duplicate data entry. Wide dissemination of EHR-integrated CDS has been difficult, however, with a major challenge being the lack of standards-based interoperability for CDS deployment across health systems and EHR platforms.[28–30]

Recent efforts have advanced the vision of standards-based CDS interoperability across EHR platforms,[31–40] and the project team has been at the forefront of these efforts. Table 2 on the next page summarizes these efforts and our role. As detailed in Aim 1, we will enable CDS interoperability leveraging the standards developed in these efforts, including the Clinical Quality Framework (CQF) standards for CDS and electronic clinical quality measure (eCQM) interoperability developed with PI Kawamoto's leadership and through the sponsorship of CMS and the US Office of the National Coordinator for Health IT (ONC). A summary of CDS interoperability standards is also available in an AHRQ-sponsored Webinar on the topic given by PI Kawamoto.[41]

This project will enable widespread CDS to optimize lung cancer LDCT screening and provide a model for widely disseminating other evidence-based CDS. We will adapt the stand-alone Decision Precision tool into a standards-based CDS tool that is fully integrated with the EHR (Aim 1), integrate the tool with multiple EHR systems and widely disseminate the tool (Aim 2), and evaluate the reach and impact of the tool (Aim 3). Because the approach will be scalable using standards-based approaches, and because use of the tool could be greatly facilitated by CMS payment rules requiring SDM when initiating LDCT lung cancer

screening,[10] the tool could ultimately enable more appropriate lung cancer LDCT screening for the estimated 8.6 million US adults who meet USPSTF LDCT screening guidelines.[16] Such optimized LDCT screening could prevent as many as 10,000 lung cancer deaths annually while minimizing adverse events associated with screening.[8,13,15,16]

The proposed SDM approach can be readily adapted to other conditions. As such, this project will provide a template for the widespread implementation of other evidence-based findings through CDS-enabled SDM.

Table 2. Notable Initiatives Related to CDS Interoperability and Project Team Role

Initiative	Description	Project Team Role
AHRQ PCCDS (formerly PCOR CDS) Learning Network[36]	Collaboration community for learning how best to translate evidence-based research findings to improve patient outcomes via patient-centered CDS	Steering Committee Member (PI Kawamoto)
AHRQ CDS Connect[37]	Project to develop repository and dissemination channels for standards-based CDS artifacts	Work group member for CDS repository design and implementation (PI Kawamoto)
Health Level 7 International (HL7)	Leading standards development organization for health IT internationally	Board Member (PI Kawamoto) and Co-Chairs of CDS Work Group (PI Kawamoto, co-I Del Fiol)
ONC/CMS CQF initiative[31]	Effort to develop and validate harmonized HL7 interoperability standards for CDS and eCQM	Co-Initiative Coordinator (PI Kawamoto)
ONC Health eDecisions (HeD)[38]	Predecessor effort to CQF that developed and validated HL7 CDS interoperability standards	Initiative Coordinator (PI Kawamoto)
US Health IT Advisory Committee (HITAC)[42]	Committee established by 21st Century Cures Act to provide counsel to National Coordinator for Health IT	Co-chair of Interoperability Standards Priorities Task Force (PI Kawamoto)
National Academy of Medicine CDS	Effort to develop action plan for interoperable, effective CDS at scale[43]	Work Group Lead for Interoperable CDS Content (PI Kawamoto)
SMART on FHIR[39] and CDS Hooks[44]	Efforts to embed interoperable apps and CDS services within EHRs via OAuth and FHIR standards	Active implementers of standards-based CDS solutions in production settings (PI Kawamoto)
OpenCDS[40]	Open-source, freely-available implementation of CDS and eCQM interoperability standards	Founder and lead (PI Kawamoto)

LDCT is currently the only recommended screening option for the lung cancer screening.[45] Chest Radiographs have been evaluated in a large randomized controlled trial and found to be ineffective.[46] If a patient chooses to forego LDCT screening, the provider is expected to support this decision. In such cases, the provider may recommend risk-mitigating strategies such as smoking cessation, avoiding second-hand smoke, avoiding asbestos exposure, and improving diet and exercise. Respecting the patient's autonomy is especially important when the balance of benefits and harms makes the screening preference-sensitive. In such cases, the provider is expected to explain that both screening and not screening are reasonable. While the decision to screen or not screen will ultimately be up to the patient, if a patient is not healthy enough to undergo curative lung resection or has limited life-expectancy due to other comorbidities, that patient is unlikely to benefit from lung cancer screening, and the provider would be expected to recommend against screening. The provider may also recommend against screening if the balance of harms versus benefits is unfavorable, or if the patient does not meet USPSTF criteria for lung cancer screening. In any case, the decision will ultimately be up to the patient.

References

1. Siegel RL, Miller KD, Jemal A. Cancer statistics, 2018. *CA Cancer J Clin*. 2018;68(1):7-30. doi:10.3322/caac.21442

2. Aberle DR, Adams AM, Berg CD, et al. Reduced Lung-Cancer Mortality with Low-Dose Computed Tomographic Screening. *N Engl J Med*. 2011;365(5):395-409. doi:10.1056/nejmoa1102873

3. Moyer VA. Screening for Lung Cancer: U.S. Preventive Services Task Force Recommendation Statement. *Ann Intern Med*. 2014;160(5):330-338. doi:10.7326/m13-2771

4. US Preventive Services Task Force. Final recommendation statement: lung cancer screening (2013). Published 2013. Accessed June 2, 2021. https://uspreventiveservicestaskforce.org/uspstf/recommendation/lung-cancer-screening-december-2013

5. US Preventive Services Task Force. Final recommendation statement: lung cancer screening (2021). Accessed June 2, 2021. https://uspreventiveservicestaskforce.org/uspstf/recommendation/lung-cancer-screening

6. Meza R, Jeon J, Toumazis I, et al. Evaluation of the Benefits and Harms of Lung Cancer Screening with Low-Dose Computed Tomography: Modeling Study for the US Preventive Services Task Force. *JAMA - J Am Med Assoc*. 2021;325(10):988-997. doi:10.1001/jama.2021.1077

7. Tammemägi MC, Katki HA, Hocking WG, et al. Selection Criteria for Lung-Cancer Screening. *N Engl J Med*. 2013;368(8):728-736. doi:10.1056/nejmoa1211776

8. Kovalchik SA, Tammemagi M, Berg CD, et al. Targeting of Low-Dose CT Screening According to the Risk of Lung-Cancer Death. *N Engl J Med*. 2013;369(3):245-254. doi:10.1056/nejmoa1301851

9. Bach PB, Kattan MW, Thornquist MD, et al. Variations in Lung Cancer Risk Among Smokers. *JNCI J Natl Cancer Inst*. 2003;95(6):470-478. doi:10.1093/jnci/95.6.470

10. Centers for Medicare and Medicaid Services. Decision memo for screening for lung cancer with low dose computed tomography. Published 2015. Accessed August 29, 2019. https://www.cms.gov/medicare-coverage-database/details/nca-decision-memo.aspx?NCAId=274.

11. O'Connor AM. Validation of a Decisional Conflict Scale. *Med Decis Mak*. 1995;15(1):25-30. doi:10.1177/0272989x9501500105

12. Charles C, Gafni A, Whelan T. Shared decision-making in the medical encounter: What does it mean? (or it takes at least two to tango). *Soc Sci Med*. 1997;44(5):681-692. doi:10.1016/s0277-9536(96)00221-3

13. Jemal A, Fedewa SA. Lung Cancer Screening With Low-Dose Computed Tomography in the United States—2010 to 2015. *JAMA Oncol*. 2017;3(9):1278. doi:10.1001/jamaoncol.2016.6416

14. Pham D, Bhandari S, Oechsli M, Pinkston CM, Kloecker GH. Lung cancer screening rates: Data from the lung cancer screening registry. *J Clin Oncol*. 2018;36(15_suppl):6504. doi:10.1200/jco.2018.36.15_suppl.6504

15. Caverly TJ, Cao P, Hayward RA, Meza R. Identifying Patients for Whom Lung Cancer Screening Is Preference-Sensitive. *Ann Intern Med*. 2018;169(1):1. doi:10.7326/m17-2561

16. Ma J, Ward EM, Smith R, Jemal A. Annual number of lung cancer deaths potentially avertable by screening in the United States. *Cancer*. 2013;119(7):1381-1385. doi:10.1002/cncr.27813

17. Kinsinger LS, Anderson C, Kim J, et al. Implementation of Lung Cancer Screening in the Veterans Health Administration. *JAMA Intern Med*. 2017;177(3):399. doi:10.1001/jamainternmed.2016.9022

18. Moyer A, Salovey P. Patient participation in treatment decision making and the psychological consequences of breast cancer surgery. *Womens Health*. 1998;4(2):103-116. Accessed August 29, 2019. http://www.ncbi.nlm.nih.gov/pubmed/9659000

19. Street RL, Voigt B. Patient Participation in Deciding Breast Cancer Treatment and Subsequent Quality of Life. *Med Decis Mak*. 1997;17(3):298-306. doi:10.1177/0272989x9701700306

20. Brian Haynes R, Ann McKibbon K, Kanani R. Systematic review of randomised trials of interventions to assist patients to follow prescriptions for medications. *Lancet*. 1996;348(9024):383-386. doi:10.1016/s0140-6736(96)01073-2

21. Agency for Healthcare Research and Quality. Lung cancer screening: a clinician's checklist. Published 2016. Accessed August 29, 2019. https://effectivehealthcare.ahrq.gov/tools-and-resources/patient-decision-aids/lung-cancer-screening/clinicians-checklist/

22. Memorial Sloan Kettering Cancer Center. Lung cancer screening decision tool. Published 2016. Accessed August 29, 2019. http://nomograms.mskcc.org/Lung/Screening.aspx

23. American Lung Association. Lung cancer screening saves lives [online patient decision aid]. Published 2016. Accessed August 29, 2019. https://www.lung.org/our-initiatives/saved-by-the-scan/?utm_source=domain&utm_medium=redirect&utm_campaign=saved_by_the_scan

24. University of Michigan. Lung cancer CT screening: should I get screened? Published 2016. Accessed August 29, 2019. https://shouldiscreen.com/English/home

25. Caverly T. Selecting the Best Candidates for Lung Cancer Screening. *JAMA Intern Med*. 2015;175(6):898. doi:10.1001/jamainternmed.2015.1235

26. Kawamoto K, Houlihan CA, Balas EA, Lobach DF. Improving clinical practice using clinical decision support systems: a systematic review of trials to identify features critical to success. *BMJ*. 2005;330(7494):765. doi:10.1136/bmj.38398.500764.8f

27. Bates DW, Kuperman GJ, Wang S, et al. Ten Commandments for Effective Clinical Decision Support: Making the Practice of Evidence-based Medicine a Reality. *J Am Med Informatics Assoc*. 2003;10(6):523-530. doi:10.1197/jamia.m1370

28. Osheroff JA, Teich JM, Middleton B, Steen EB, Wright A, Detmer DE. A Roadmap for National Action on Clinical Decision Support. *J Am Med Informatics Assoc*. 2007;14(2):141-145. doi:10.1197/jamia.m2334

29. Sittig DF, Wright A, Osheroff JA, et al. Grand challenges in clinical decision support. *J Biomed Inform*. 2008;41(2):387-392. doi:10.1016/j.jbi.2007.09.003

30. Kawamoto K, Del Fiol G, Lobach DF, Jenders RA. Standards for Scalable Clinical Decision Support: Need, Current and Emerging Standards, Gaps, and Proposal

for Progress. *Open Med Inform J*. 2010;4(1):235-244. doi:10.2174/1874431101004010235

31. Clinical Quality Framework Initiative. Clinical Quality Framework homepage. Accessed August 29, 2019. https://oncprojecttracking.healthit.gov/wiki/display/TechLabSC/CQF+Home

32. Health Level 7. HL7 Guideline Appropriate Ordering FHIR Implementation Guide. Accessed August 29, 2019. https://www.hl7.org/fhir/2016Jan/gao/gao.html

33. Healthcare Services Platform Consortium. The Healthcare Innovation Ecosystem. Published 2016. Accessed August 29, 2019. https://healthservices.atlassian.net/wiki/display/HSPC/Healthcare+Services+Platform+Consortium

34. Middleton B. The clinical decision support consortium. *Stud Health Technol Inform*. 2009;150:26-30. Accessed August 30, 2019. http://www.ncbi.nlm.nih.gov/pubmed/19745260

35. Office of the National Coordinator for Health IT. Certification of Health IT. Published online 2018. https://www.healthit.gov/topic/certification-ehrs/certification-health-it

36. AHRQ Patient-Centered CDS Learning Network. Patient-Centered CDS Learning Network Home Page. https://pccds-ln.org/

37. Agency for Healthcare Research and Quality. CDS Connect. Accessed August 30, 2019. https://cds.ahrq.gov/cdsconnect

38. Heatlh eDecsions Initiative. Health eDecisions homepage. Accessed August 30, 2019. https://oncprojecttracking.healthit.gov/wiki/display/TechLabSC/Health+eDecisions.

39. Mandel JC, Kreda DA, Mandl KD, Kohane IS, Ramoni RB. SMART on FHIR: A standards-based, interoperable apps platform for electronic health records. *J Am Med Informatics Assoc*. 2016;23(5):1-10. doi:10.1093/jamia/ocv189

40. OpenCDS Home. Open Clinical Decision Support Tools and Resources. Accessed August 21, 2018. http://www.opencds.org/

41. AHRQ Patient-Centered CDS Learning Network. Patient-Centered CDS Learning Network Webinars. Published 2017. Accessed August 30, 2019. https://pccds-ln.org/webinars

42. Office of the National Coordinator for Health IT. Health IT Advisory Committee. Published 2018. Accessed August 30, 2019. https://www.healthit.gov/hitac/

43. Tcheng JE, Bakken S, Bates DW, et al. *The Learning Health System Series. Optimizing Strategies for Clinical Desicion Support. Summary of a Meeting Series*. National Academy of Medicine; 2017. https://lccn.loc.gov/2017055006

44. CDS Hooks Overview. Accessed September 23, 2020. http://cds-hooks.org/

45. Who Should Be Screened for Lung Cancer? Centers for Disease Control and Prevention. Published 2018. Accessed October 10, 2019. https://www.cdc.gov/cancer/lung/basic_info/screening.htm

46. Oken MM, Hocking WG, Kvale PA, et al. Screening by chest radiograph and lung cancer mortality: The Prostate, Lung, Colorectal, and Ovarian (PLCO) randomized trial. *JAMA - J Am Med Assoc*. 2011;306(17):1865-1873. doi:10.1001/jama.2011.1591

Purpose and Objectives

The purpose of this project is to increase appropriate low-dose computed tomography (LDCT) lung cancer screening through the development and wide dissemination of patient-centered clinical decision support (CDS) tools that (1) are integrated with the electronic health record (EHR) and clinical workflows, (2) prompt for shared decision making (SDM) when patients meet screening criteria, and (3) enable effective SDM using individually-tailored information on the potential benefits and harms of screening. The study will promote standard of care that is endorsed by the Centers for Medicare & Medicaid Services (CMS) and the US Preventive Services Task Force (USPSTF).The US Preventive Services Task Force (USPSTF) provided a grade B recommendation in 2013 that annual LDCT lung cancer screening be offered to patients 55 to 80 years old with a 30+ pack-year smoking history who are current smokers or quit in the last 15 years. In March 2021, the USPSTF updated its recommendation to lower the eligibility age to 50 and the smoking requirement to 20+ pack-years.

This project is supported both operationally and by an Agency for Healthcare Research and Quality (AHRQ) R18 grant.

The EHR-based CDS tools are being developed by the University of Utah ReImagine EHR team, directed by Dr. Kensaku Kawamoto. Within University of Utah Health, Dr. Kawamoto is also Associate Chief Medical Information Officer and Director of the health system's Knowledge Management and Mobilization unit. Dr. Kawamoto's team receives operational funding from University of Utah Health to develop EHR-based solutions to improve patient care and the provider experience. Grant funding such as the current AHRQ R18 grant enable this operational team to allocate more resources to the optimal design, development, and implementation of these software tools, as well as to undertake more rigorous intervention design and evaluation procedures that would not be possible in the context of operational quality improvement (QI).

This project will leverage Decision Precision (https://share.lungdecisionprecision.com/), a validated Web-based tool for LDCT SDM developed at the Veterans Health Administration, as well as an initial version of Decision Precision+, an EHR-integrated version of the tool which can be accessed directly in the EHR and auto-populate relevant patient data in the tool instead of requiring manual data entry. An initial version of Decision Precision+ was developed and made accessible within the Epic EHR shortly after the AHRQ grant was submitted for operational QI purposes. However, due to feedback from physician leaders that the system needed to be streamlined for optimal use in busy primary care settings, the tool's availability was never advertised and no CDS prompts were introduced to encourage its use. Consequently, tool access has been minimal to date.

This study seeks to build upon our existing work in CDS and SDM to promote appropriate LDCT lung cancer screening as recommended by the USPSTF and CMS. Listed below are the three study aims, as well as the human subjects research associated with each aim.

Aim 1. System Design and Development

Design and develop a standards-based CDS tool for lung cancer screening SDM that is integrated with the EHR and can be effectively used in busy primary care settings (Decision Precision+); design and develop CDS tools for optimally integrating the tool into clinical workflows; and advance underlying standards and their adoption. Information for user-centered design and workflow assessments will be collected through cognitive work analysis interviews with clinicians at University of Utah Health. Human subjects research for this aim will consist of the following:

- *Pre-Implementation Provider and Staff Interviews*

Aim 2. Implementation Trial and Iterative System Refinement

Conduct a pragmatic implementation trial of Decision Precision+ and associated CDS tools within University of Utah Health. Make Decision Precision+ available to other institutions through app stores, and also share associated CDS tools. There will be no randomization, and the actual clinical trial will be preceded by a pilot implementation to ensure that the tools are useable and compatible with clinical workflows. The study population for the clinical trial will be primary care patients at University of Utah Health who are eligible for or potentially eligible for LDCT screening and the associated SDM, and the initial intervention will include availability of Decision Precision+ combined with CDS prompts to promote its appropriate use. The intervention will be enhanced based on feedback, with anticipated enhancements including prompting medical assistants to collect required smoking history information from potentially eligible patients, as well as prompting patients to consider LDCT screening and SDM via the personal health record. Human subjects research for this aim will consist of the following:

- *Provider and Staff Participation in Pilot Implementation*

- *Patient Participation in Pilot Implementation*

- Provider and Staff Participation in Clinical Trial

- Patient Participation in Clinical Trial

- Post-implementation Provider and Staff Interviews

Aim 3. Evaluation

Evaluate the impact of the CDS tool, including for adoption, clinical impact, and financial impact. We will conduct an interrupted time series (ITS) study of the implementation trial; focus groups and surveys with patients, staff, and providers; and evaluation of the resource costs associated with implementation. Human subjects research for this aim will consist of the following:

- Patient Outcomes Assessment

- Provider and Staff App Usage Assessment

- Provider and Staff Surveys

- Patient Surveys

- Provider and Staff Focus Groups

- Patient Focus Groups

- Stakeholder Interviews

Study Population

Age of Participants: 18+

Sample Size:
At Utah:
All Centers: up to 21,000 patients

Inclusion Criteria:

Aim 1. Design

Pre-Implementation Provider and Staff Interviews: Up to 36 providers and staff members will be included in the interviews. Providers and staff will be eligible for the interviews if they provide care related to lung cancer screening for the targeted patient population at a University of Utah primary care clinic.

Aim 2. Implementation

Provider and Staff Participation in Pilot Implementation: Up to 200 providers and staff will serve as pilot users of the intervention. Providers and staff will be eligible if they volunteer to serve as pilot users or provide care related to lung cancer screening for the targeted patient population at a pilot clinic.

Patient Participation in Pilot Implementation: We anticipate up to 2,000 patients may be a part of the pilot implementation, with the intervention available to support their care. This population will consist of patients eligible for, or potentially eligible for, LDCT lung cancer screening according to USPSTF guidelines, who are seen by a pilot user of the intervention. The inclusion criteria for pilot implementation are (i) >= 55 years and <= 80 years old at the time of the visit; (ii) does not already have lung cancer; and (iii) meets 2013 USPSTF smoking criteria for LDCT screening (30+ pack-year smoking history and current smoker or quit in the past 15 years) or may meet the criteria if a complete smoking history were taken.

Provider and Staff Participation in Clinical Trial: Up to 1,000 providers and staff in primary care will be included in the clinical trial. Providers and staff will be eligible if they provide care related to lung cancer screening for the targeted patient population at a University of Utah primary care clinic.

Patient Participation in Clinical Trial: We anticipate up to 19,000 primary care patients will be enrolled, with the intervention available to support their care. Patients will be eligible for inclusion in the trial if they receive care at University of Utah primary care clinics. The study population will consist of patients eligible for, or potentially eligible for, LDCT lung cancer screening according to USPSTF guidelines, who are seen at one of these clinics. The inclusion criteria for phase 1 are (i) >= 55 years and <= 80 years old at the time of the visit; (ii) does not already have lung cancer; and (iii) meets 2013 USPSTF smoking criteria for LDCT screening (30+ pack-year smoking history and current smoker or quit in the past 15 years) or may meet the criteria if a complete smoking history were taken. The inclusion criteria for phase 2 are (i) >= 50 years and <= 80 years old at the time of the visit; (ii) does not already have lung cancer; and (iii) meets 2021 USPSTF smoking criteria for LDCT screening (20+ pack-year smoking history and current smoker or quit in the past 15 years) or may meet the criteria if a complete smoking history were taken.

Post-Implementation Provider and Staff Interviews: Up to 36 providers and staff who participated in the pilot implementation or clinical trial will be included in the interviews.

Aim 3. Evaluation

Patient Outcomes Assessment: up to 21,000 patients will be enrolled. Eligibility criteria are the same as for participation in the pilot implementation and clinical trial.

Provider and Staff App Usage Assessment: up to 1,200 providers and staff will be enrolled. Eligibility criteria are the same as for participation in the pilot implementation and clinical trial.

Provider and Staff Surveys: Up to 400 providers and staff from University of Utah primary care clinics will be surveyed regarding the intervention. Providers and staff will be eligible if they have interacted with the intervention through the pilot implementation or clinical trial.

Patient Surveys: Up to 200 patients from University of Utah primary care clinics will be surveyed regarding the intervention. Patients will be eligible if the intervention was used in their care and they speak English.

Provider and Staff Focus Groups: Up to 36 providers and staff will be enrolled in focus groups to evaluate the intervention as well as explore implementation and adoption issues with CDS, SDM, and guideline-focused care in general. Eligibility criteria will be the same as for the provider and staff surveys.

Patient Focus Groups: Up to 18 patients will be enrolled in focus groups to evaluate the intervention as well as explore implementation and adoption issues with CDS, SDM, and guideline-focused care in general. Eligibility criteria will be the same as for the patient surveys.

Stakeholder Interviews: Up to 100 key stakeholders who implemented the intervention at the University of Utah or elsewhere will be interviewed with a goal of understanding barriers and facilitators to the scalable dissemination of evidence-based CDS, as well as to estimate the associated time and resource costs. Eligibility criteria will be involvement in the implementation of the intervention for clinical care purposes.

Exclusion Criteria:
None.

Design

Prospective Biomedical Intervention or Experiment

Study Procedures

Recruitment/Participant Identification Process:

Aim 1. Design

Pre-Implementation Provider and Staff Interviews: We will recruit participants in-person or via email in consultation with Dr. Michael Flynn, who oversees research and informatics for the University of Utah Community Physicians Group, and who is a co-investigator on the project. Recruitment may also take place during staff meetings or short educational presentations at clinic sites by Dr. Flynn, other research staff or other clinic directors.

Aim 2. Implementation

Provider and Staff Participation in Pilot Implementation and Clinical Trial: Because the intervention is promoting standard of care, we will base our approach on how University of Utah Health operationally introduces other health IT interventions to promote standard of care. The PI is Associate Chief Medical Information Officer for the health system and routinely introduces similar interventions for operational purposes outside of a research context. We will follow this usual process, including seeking approval from relevant governance groups such as the Community Physical Group Medical Directors or the EHR Operations committee. We will seek a waiver of informed consent for individual providers and staff as described later.

Patient Participation in Pilot Implementation and Clinical Trial: We will seek a waiver of informed consent for targeted patients as described later.

Post-Implementation Provider and Staff Interviews: We will use the same recruitment strategies as for Aim 1.

Aim 3. Evaluation

Patient Outcomes Assessment: The recruitment approach will be the same as for the patient participation in the pilot implementation and clinical trial as described above.

Provider and Staff App Usage Assessment: The recruitment approach will be the same as for the provider and staff participation in the pilot implementation and clinical trial as described above.

Provider and Staff Surveys: Survey study participants will be recruited via email. To improve the response rate, we may send up to three reminders. Additionally, we may recruit through targeted in-person distribution of the surveys by research team members.

Patient Surveys: Survey study participants will be recruited electronically or via postal mail.

Provider and Staff Focus Groups: We will ask in the surveys above if participants are potentially interested in the focus groups. We will recruit from those respondents who answer positively to this question. If needed, additional participants will be recruited in-person or via email.

Patient Focus Groups: We will ask in the surveys above if participants are potentially interested in the focus groups. We will recruit from those respondents who answer positively to this question.

Stakeholder Interviews: We will recruit participants via email.

Data Quality Assurance: Because the intervention is promoting standard of care, we will base our approach on how University of Utah Health operationally introduces other health IT

interventions to promote standard of care. As part of standard quality assurance procedures, we will assess smoking history and comorbidities data for all adults who had an outpatient visit in University of Utah Health primary care clinic.

Informed Consent:

Description of location(s) where consent will be obtained:

For interviews and focus groups, verbal consent will be obtained where the interviews and focus groups are conducted, which are expected to be at the clinics. For surveys, proceeding with the survey will constitute consent. This is expected to take place at the clinics or at the respondents' homes.

Description of the consent process(es), including the timing of consent:

Aim 1. Design Pre-Implementation Provider and Staff Interviews: Oral informed consent will be obtained at the beginning of the interview process following participant review of a Consent Cover Letter. See item 8 for details. Aim 2. Implementation Provider and Staff Participation in Pilot Implementation: For the pilot implementation, a waiver of informed consent is sought following assent of the pilot users or the clinical leadership (see Waiver request). Patient Participation in Pilot Implementation: A waiver of informed consent is sought following assent of assent of the pilot users or the clinical leadership (see Waiver request). Provider and Staff Participation in Clinical Trial: A waiver of informed consent is sought following assent of the clinical leadership (see Waiver request). Patient Participation in Clinical Trial: A waiver of informed consent is sought following assent of the clinical leadership (see Waiver request). Post-Implementation Provider and Staff Interviews: For provider and staff interviews, verbal informed consent will be obtained at the beginning of the interview process. Aim 3. Evaluation Patient Outcomes Assessment: A waiver of informed consent is sought as for the Patient Participation in the Pilot Implementation and Clinical Trial. Provider and Staff App Usage Assessment: A waiver of informed consent is sought as for the Provider and Staff Participation in the Pilot Implementation and Clinical Trial. Provider and Staff Surveys: Electronic informed consent will be obtained at the beginning of the survey. See item 8 for details. Patient Surveys: Proceeding with the survey will constitute consent. See item 8 for details. Provider and Staff Focus Groups: Oral informed consent will be obtained at the beginning of the focus groups following participant review of a Consent Cover Letter. See item 8 for details. Patient Focus Groups: Oral informed consent will be obtained at the beginning of the focus groups following participant review of a Consent Cover Letter. See item 8 for details. Stakeholder Interviews: Oral informed consent will be obtained at the beginning of the interviews following participant review of a Consent Cover Letter. See item 8 for details.

Requested Waivers/Alterations of Consent:

Waiver of Informed Consent	Request for waiver of consent for providers and staff to be exposed to EHR-based intervention facilitating lung cancer shared decision making for patients meeting USPSTF eligibility criteria for this clinical service
Waiver of Informed Consent	Request for waiver of consent for patient to have their providers and staff to have access to the IT intervention promoting

standard of care, and to evaluate their data to assess intervention impact.

Procedures:

Listed below are the procedures for the human subjects research aspects of this project. Procedures for activities outside of humans subjects research are not listed, such as engagement with standards development organizations to advance relevant health IT standards; software development, implementation and monitoring undertaken to fulfill clinical operations and operational QI responsibilities rather than for research purposes; and making the software developed in this project available in EHR app stores.

Aim 1. System Design and Development

Pre-Implementation Provider and Staff Interviews: During each interview, the interviewer will ask the participant to recall a recent relevant patient and provide a 1) summary, 2) timeline, and 3) a deepened description. After each interview, we will display the intervention or a mock-up (if not yet fully implemented) and conduct usability assessments. The interviews will be coded and modeled through consensus by members of the research team. The results of the interviews will be used to guide refinement of the intervention. Interviews are expected to take approximately 45 minutes to 1 hour, taking place outside of normal work hours. Audio and screen recordings will be made of the interviews where appropriate with participant consent. Screen recording tools may include eye tracking. We may record the screen, eye movements, and audio to analyze the app use by the participant. Audio-recordings may be transcribed.

Aim 2. Implementation

Provider and Staff Participation in Pilot Implementation: To account for real-world conditions, the interventions will be pilot tested by providers and staff who volunteer to serve as pilot users or provide care related to lung cancer screening for the targeted patient population at a pilot clinic.

Patient Participation in Pilot Implementation: Patients will be included in the pilot implementation if they meet the pilot implementation inclusion criteria as described earlier.

Provider and Staff Participation in Clinical Trial: The project team will work with leaders in primary care at the University of Utah to identify how best to incorporate the intervention into clinical workflows. Based on these discussions and following assent by the clinical leaders, we anticipate that we will visit each clinic to describe the intervention, conduct training, and answer questions. It is anticipated that usual clinical staff meetings will be leveraged for this purpose. Providers and designated staff will have access to the intervention in the EHR. Providers and staff will be free to follow or disregard the intervention's suggestions as they see fit.

There will not be randomization in this study. The intervention will be rolled out systemwide.

The intervention will consist of the following core items:

- An EHR-integrated shared decision making (SDM) tool for providing information on the risks and benefits of lung cancer screening through low-dose computed tomography (LDCT) testing. An initial version of this tool (Decision Precision+) was previously approved for operational clinical use outside of this research study. A current version of the standalone version of the SDM tool is available at https://share.lungdecisionprecision.com/.

- Reminders in the EHR to offer SDM for LDCT for patients eligible for lung cancer screening according to USPSTF guidelines. Epic "Health Maintenance" reminders for this purpose were also previously approved for operational clinical care through usual clinical governance channels. Additional reminders may be provided using other EHR-based mechanisms, including through an Epic-integrated disease management and health maintenance system that is also already available in the Epic EHR system at the University of Utah.

Based on feedback received from users, it is anticipated that at least one cycle of improvement will be released into the intervention during the course of the pragmatic implementation trial. For study power analysis purposes, we anticipate one improvement release approximately 9 months into the trial, with the trial anticipated to last 18 months. We anticipate the intervention will remain as a part of routine clinical care following the trial. In addition to these major enhancements, less significant updates or bug fixes, if any, will be released as clinically required. All major enhancements will be introduced with the support and approval of the appropriate governance group, such as the Community Physicians Group Medical Directors or the EHR Operations committee. While the nature of the major intervention enhancements will be dependent on user feedback, we currently anticipate that one or more of the following will be introduced as the enhancements:

- Providing eligible patients with the recommendation to undergo shared decision making for lung cancer screening. This is already done for many USPSTF recommended preventive care reminders. We anticipate that we will provide these care reminders through the Epic MyChart personal health record portal.

- Introducing Epic Health Maintenance prompts for medical assistants to collect detailed smoking history for patients who may be eligible for screening (age 55 to 80 with a history of smoking and no lung cancer diagnosis) but who lack the detailed smoking history data required to make a complete determination (e.g., pack-years of smoking and years since quit smoking).

- Facilitating patient education outside of the clinic visit, e.g., through printing out the shared decision making materials during the visit, for patient review prior to a follow-up discussion with the provider at a subsequent visit.

Following the roll-out, clinic leaders may receive periodic feedback on use of the intervention and LDCT screening rates, including comparisons to other clinics. Clinic leaders may be given user-level data so that outlier performances can be addressed if desired. Activities for facilitating implementation may include regular email, phone, or in-person communication with clinic leaders to discuss CDS adoption and address barriers to adoption; identify "implementation pearls"; and share these "pearls" with other clinics. We and others have shown that such facilitation activities improve the adoption and impact of practice change interventions. Contact frequency will be determined by workflow adoption, with more intensive follow-up directed to clinics with lower adoption rates. We will maintain a log of interactions to identify common issues, and issues will be prioritized and addressed.

Patient Participation in Clinical Trial: Patients will be included in the clinical trial if they meet the clinical trial inclusion criteria described earlier.

Post-Implementation Provider and Staff Interviews: Early formative interviews will be conducted with care team members at the pilot clinic, and potentially other intervention clinics, as the users respond to the intervention. The purpose is to examine impact on workflow, uncover any usability problems, refine implementation procedures, and identify any training needs. Interviews are expected to take approximately 45 minutes to 1 hour. Audio and screen recordings will be made of the interviews where appropriate with participant consent. Audio-recordings may be transcribed. Screen recording tools may include eye tracking. Some

screen captures may include actual patient data. We may record the screen, eye movements, and audio to analyze intervention use by the participant for later analysis or for participants to comment on their thought process as they review their own recordings.

Aim 3. Evaluation

Patient Outcomes Assessment: Data for analysis will be extracted from the University of Utah data warehouse and system logs. Outcome measures are described under section 9 below. A PhD-level statistician co-investigator will oversee the statistical analyses. Patients for whom the intervention was used prior to the clinical trial will be excluded from the main clinical trial analyses.

Provider and Staff App Usage Assessment: The same approach as for the Patient Outcomes Assessment will be used.

Provider and Staff Surveys: Providers and staff will be surveyed regarding their satisfaction with the intervention using the System Usability Scale (SUS), with supplemental questions on barriers and facilitators to usage as well as questions on the impact on workflow and workload. We will also ask whether respondents are interested in the focus groups described next. Providers and staff will be surveyed electronically, with invitations sent via email.

Patient Surveys: Patients will be surveyed electronically and/or on paper with invitations, surveys, and pre-paid return envelopes sent by mail. Patients will be surveyed regarding their screening preferences, knowledge, preference for SDM, decisional conflict, and satisfaction with provider communication. We will also ask whether respondents are interested in the focus groups described next.

Provider and Staff Focus Groups: The focus groups will evaluate the intervention specifically, as well as explore the implementation and adoption issues with CDS, SDM, and guideline-focused care in general. Methods suggested by Krueger and associates will be utilized, including a prepared script (Introduction, Overview, Ground Rules, and Questions), use of non-threatening group processes, and homogenous groups. We will focus on facilitators and barriers to CDS adoption. Each focus group is expected to take 1-1.5 hours. Discussions will be recorded and may be transcribed.

Patient Focus Groups: The same methods as for the Provider and Staff Focus Groups will be used.

Stakeholder Interviews: We will interview key stakeholders at each of the implementation sites, including dissemination sites in other healthcare systems, with a goal of understanding barriers and facilitators to the scalable dissemination of evidence-based CDS, as well as to estimate the associated time and resource costs. To facilitate cost estimation, stakeholders will be asked to keep track of significant resource expenditures.

Data Quality Assurance: Because the intervention is promoting standard of care, we will base our approach on how University of Utah Health operationally introduces other health IT interventions to promote standard of care. As part of standard quality assurance procedures, we will assess all adults who had an outpatient visit in University of Utah Health primary care. Data for smokers would be analyzed to find inaccuracies and new estimates for lung cancer risk and eligibility for lung cancer will be produced.

Our grant application matches the ERICA application in the following areas: study design, study population, study objectives and goals, and study test interventions and procedures. Surveys, interview guides, and focus group guides have been uploaded in the Document and Attachment section. In case of any edits, all of these materials will be submitted to the IRB via amendment for review and approval before they are used with participants.

Procedures performed for research purposes only:

Statistical Methods, Data Analysis and Interpretation

Aim 1. Design

Pre-Intervention Provider and Staff Interviews: Records will be analyzed using qualitative analysis software (such as ATLAS or NVivo), which can integrate transcripts, pictures, memos and other materials. We will use procedures recommended by Patton and others that focus on developing coding protocols to highlight issues, problems, and potential recommendations.

Aim 2. Implementation

*Pilot Implementation and Clinical Trial: s*ee below for analyses for the pilot implementation and clinical trial.

Post-Implementation Provider and Staff Interviews: The purpose is to examine impact on workflow, uncover any usability problems, refine implementation procedures, and identify any training needs. We will use qualitative analyses procedures similar to Aim 1.

Aim 3. Evaluation

Patient Outcomes Assessment: The primary outcome is the proportion of patients eligible for LDCT screening per USPSTF guidelines receiving LDCT screening. Planned secondary outcomes are the expected number of lung cancer deaths prevented given the risk profiles of individuals screened with LDCT, the expected number of major complications given the risk profiles of individuals screened with LDCT, and, if approved by the University of Utah Value Driven Outcomes research committee, the operating margin attributable to study patients during study period (total revenues minus total cost). Data for the operating margin analysis will leverage the Value Driven Outcomes value analysis tool co-developed by PI Kawamoto to determine the true costs of care.

We will report the number of patients and patient characteristics for each phase of the interrupted time series (ITS) study. We anticipate a 12-month baseline phase, a 9-month intervention phase, and a 9-month enhanced intervention phase which incorporates improvements made to the CDS intervention based on initial experience. We will conduct a segmented regression analysis to fit the monthly series and assess the extent to which the intervention was associated with changes in outcome measures. The study phases constitute three segments of regression models with monthly time points. We will use the segmented least squares approach with parameters for intercept, baseline trend, and changes in the level

and trend after the intervention. We will start with assuming a linear trend line within each segment. Nonlinearity trends will be explored if the linear assumption is violated. We will use the Durbin Watson statistic as a measure of autocorrelation and test up to six-order autocorrelation. Potential confounding is limited to covariates associated with the outcome that change at the time of the intervention. We will test for confounding due to changes in study population characteristics such as age, gender and race. Statistical significance will be defined at alpha = 0.05.

To mitigate the impact of pandemic COVID19, we will make the following two modifications in the statistical analysis plan. First, we will redefine the baseline phase so that it has no overlap with the lockdown period, because all the non-essential medical visits have been cancelled during this period. Secondly, we anticipate the patient characteristics and composition during the intervention phase and even enhanced intervention phase might be different from the baseline phase which is observed before the pandemic. We will compare the distributions of patient characteristics across the three phases of the ITS study. If significant discrepancies are observed in the patient characteristics and composition, we will conduct propensity score modeling and use a matching or weighting approach to create a comparable study cohort in each phase before running the originally proposed segmented regression analysis. If any lock-down periods coincide with the intervention phases, we may remove visits occurring during these periods from the analysis.

To estimate statistical power, we followed Rozario, Moore and McWilliams' approach, and used the statistical software SAS. We assumed the proportion of eligible patients receiving LDCT screening, the primary outcome in the study, will increase from the current 6.4% to at least 12.8% during the intervention phase and 20% during the enhanced intervention phase. Empirical EHR data indicate at least 900 patients per month will meet USPSTF LDCT eligibility criteria throughout the study period. With this conservative monthly sample estimation and the length of time at each phase, we would have more than 95% power to detect the hypothesized differences in the primary outcomes at different phases. All the estimated statistical powers were calculated based on two-sided tests with a significance level of alpha = 0.05.

Provider and Staff App Usage Assessment: Utilization metrics will include the number of clinics, providers, and other empowered staff utilizing the intervention.

Provider and Staff Surveys: Descriptive data analysis will be conducted and aggregated data will be compared across groups.

Patient Surveys: The same methods will be used as for the provider and staff surveys.

Provider and Staff Focus Groups: Records will be analyzed using qualitative analysis software (such as ATLAS or NVivo) which can integrate transcripts, pictures, memos and other materials. We will use procedures recommended by Patton and others that focus on developing coding protocols to highlight issues, problems, and potential recommendations.

Patient Focus Groups: The same methods will be used as for the provider and staff focus groups.

Stakeholder Interviews: The same methods will be used as for the provider and staff focus groups.

Data Quality Assurance: Data for smokers would be analyzed to find inaccuracies and new estimates for lung cancer risk and eligibility for lung cancer will be produced. We will use smoking history data entered in the EHR in the designated structured fields. Detailed smoking history could be recorded in 5 fields: smoking status, packs per day, years smoked, smoking start date and smoking quit date. In our system, which uses the default EHR configuration for smoking history documentation, smoking start date is usually missing as it can only be entered by patients through the patient portal. More granular smoking status data will be classified into current and former smoker categories. We will aim to develop an algorithm which could be used in the future to more accurately predict eligibility for lung cancer screening. Intermediary evaluation measures for this algorithm will include whether patients have sufficient data to calculate eligibility, pack-years and years since quit on 2021/01/01.

Electronic
RESEARCH INTEGRITY AND COMPLIANCE
THE UNIVERSITY OF UTAH

Date: Monday, February 26, 2024 8:38:55 AM

Print Close

IRB_00125797 - AM_Add Screen Recordings to the Interviews

Created: 12/19/2019 2:49 PM

PI: Kensaku Kawamoto

Submitted: 1/2/2020

Title: Design and Implementation of Scalable Decision Support and Shared Decision Making for Lung Cancer Screening

AM_00036552

1. Amendment Type

1. Amendment Introduction

Brief Description of the Study: (This will populate from original application)

The purpose of this project is to increase appropriate low-dose computed tomography (LDCT) lung cancer screening through the development and wide dissemination of patient-centered clinical decision support (CDS) tools that (1) are integrated with the electronic health record (EHR) and clinical workflows, (2) prompt for shared decision making (SDM) when patients meet screening criteria, and (3) enable effective SDM using individually-tailored information on the potential benefits and harms of screening. The study will promote standard of care that is endorsed by the Centers for Medicare & Medicaid Services (CMS) and the US Preventive Services Task Force (USPSTF). This project is supported both operationally and by an Agency for Healthcare Research and Quality (AHRQ) R18 grant.

The EHR-based CDS tools are being developed by the University of Utah ReImagine EHR team, directed by Dr. Kensaku Kawamoto. Within University of Utah Health, Dr. Kawamoto is also Associate Chief Medical Information Officer and Director of the health system’s Knowledge Management and Mobilization unit. Dr. Kawamoto’s team receives operational funding from University of Utah Health to develop EHR-based solutions to improve patient care and the provider experience. Grant funding such as the current AHRQ R18 grant enable this operational team to allocate more resources to the optimal design, development, and implementation of these software tools, as well as to undertake more rigorous intervention design and evaluation procedures that would not be possible in the context of operational quality improvement (QI).

This project will leverage Decision Precision (https://share.lungdecisionprecision.com/), a validated Web-based tool for LDCT SDM developed at the Veterans Health Administration, as well as an initial version of Decision Precision+, an EHR-integrated version of the tool which can be accessed directly in the EHR and auto-populate relevant patient data in the tool instead of requiring manual data entry. An initial version of Decision Precision+ was developed and made accessible within the Epic EHR shortly after the AHRQ grant was submitted for operational QI purposes. However, due to feedback from physician leaders that the system needed to be streamlined for optimal use in busy primary care settings, the tool’s availability was never advertised and no CDS prompts were introduced to encourage its use. Consequently, tool access has been minimal to date.

This study seeks to build upon our existing work in CDS and SDM to promote appropriate LDCT lung cancer screening as recommended by the USPSTF and CMS. Listed below are the three study aims, as well as the human subjects research associated with each aim.

Aim 1. System Design and Development

Design and develop a standards-based CDS tool for lung cancer screening SDM that is integrated with the EHR and can be effectively used in busy primary care settings (Decision Precision+); design and develop CDS tools for optimally integrating the tool into clinical workflows; and advance underlying standards and their adoption. Information for user-centered design and workflow assessments will be collected through cognitive work analysis interviews with clinicians at University of Utah Health. Human subjects research for this aim will consist of the following:

- Pre-Implementation Provider and Staff Interviews

Aim 2. Implementation Trial and Iterative System Refinement

Conduct a pragmatic implementation trial of Decision Precision+ and associated CDS tools within University of Utah Health. Make Decision Precision+ available to other institutions through app stores, and also share associated CDS tools. There will be no randomization, and the actual clinical trial will be preceded by a pilot implementation to ensure that the tools are useable and compatible with clinical workflows. The study population for the clinical trial will be primary care patients at University of Utah Health who are eligible for or potentially eligible for LDCT screening and the associated SDM, and the initial intervention will include availability of Decision Precision+ combined with CDS prompts to promote its appropriate use. The intervention will be enhanced based on feedback, with anticipated enhancements including prompting medical assistants to collect required smoking history information from potentially eligible patients, as well as prompting patients to consider LDCT screening and SDM via the personal health record. Human subjects research for this aim will consist of the following:

- Provider and Staff Participation in Pilot Implementation

- Patient Participation in Pilot Implementation

- Provider and Staff Participation in Clinical Trial

- Patient Participation in Clinical Trial

- Post-implementation Provider and Staff Interviews

Aim 3. Evaluation

Evaluate the impact of the CDS tool, including for adoption, clinical impact, and financial impact. We will conduct an interrupted time series (ITS) study of the implementation trial; focus groups and surveys with patients, staff, and providers; and evaluation of the resource costs associated with implementation. Human subjects research for this aim will consist of the following:

- Patient Outcomes Assessment

- Provider and Staff App Usage Assessment

- Provider and Staff Surveys

- Patient Surveys

- Provider and Staff Focus Groups

- Patient Focus Groups

- Stakeholder Interviews

1. **Name of Amendment:**
   Use a name that will make it easy to identify the contents of the amendment. You may use information such as the sponsor amendment number or an internal tracking number.
   Add Screen Recordings to the Interviews

2. **Type of Amendment (check all that apply):**
   Changes to study procedures:

3. **Current Status of the Study:**
   Open for Enrollment

4. **Total Number of Participants Enrolled To Date**
   At Utah: 0
   All Centers: 0

IRB_00125797 - AM_Add Screen Recordings to the Interviews **Created:** 12/19/2019 2:49 PM

PI: Kensaku Kawamoto **Submitted:** 1/2/2020

Title: Design and Implementation of Scalable Decision Support and Shared Decision Making for Lung Cancer Screening

AM_00036552
2. Description

2. Amendment Description

You have indicated that the following types of changes are being made:

Changes to study procedures:

1. **What changes are being made? List and number each change, grouping similar changes together.**

   1. Changes to procedures:

   a. We added this sentence to the procedures for pre-implementation interviews: "Audio and screen recordings will be made of the interviews where appropriate with participant consent. Screen recording tools may include eye tracking."

   b. We added this sentence to the procedures for post-implementation interviews: "Audio and screen recordings will be made of the interviews where appropriate with participant consent. Screen recording tools may include eye tracking. Some screen captures may include actual patient data."

   c. We updated pre-implementation consent docs

   2. Administrative change: inclusion criteria was updated to read "<= 80" instead of "< 80" per US Preventative Services Task Force guideline screening recommendations.

2. **Describe the reason for each of the changes described above. List and number the reasons according to the list above.**

   1. Changes to procedures:

   a. We wanted to clarify that interviews may involve audio and screen capture.

   c. We revised provider and staff interview consent docs to inform of audio recording for both provider and staff interviews, and screen capture for provider interviews

   2. Administrative change: to make sure our age range was reflected accurately in the application.

3. **How does each change described above affect participants? List and number the effects according to the above list.**

   1. Changes to procedures:

   a. Participant will be asked to give verbal consent for the audio and screen recordings in the beginning of the interview.

   2. Administrative change: No impact on participants.

4. **Will the modification(s), in the opinion of the local PI, increase or decrease the risk to participants?**
   Neither

   If the risk changes, provide justification:

5. **How will enrolled participants (current and past) be notified of this change?**

N/A - No currently enrolled participants

If Other, please explain:

6. **Which approved documents are affected by these changes?**

Consent, Parental Permission, or Assent Documents

If other, please list:

7. **Which sections of the Update Study Application are affected by these changes?**

4.6 Procedures

8. **Select all study locations that are affected by these changes.**

Prior Approved Study Controlled Locations

Site Name	Investigator Name	Covered Entity
☐ University of Utah	Kensaku Kawamoto	Study procedures will be conducted within a HIPAA Covered Entity at this site (HIPAA Privacy Rule applies)

New Sites

IRB_00125797 - AM_Add Screen Recordings to the Interviews **Created:** 12/19/2019 2:49 PM

PI: Kensaku Kawamoto **Submitted:** 1/2/2020

Title: Design and Implementation of Scalable Decision Support and Shared Decision Making for Lung Cancer Screening

AM_00036552
3. Report Forms

3. Report Forms

1. Is this amendment related to information that meets the IRB reporting policy for unanticipated problems and non-compliance?

○ Yes ● **No**

If yes, a Report Form must also be submitted and then linked to this amendment. Link the related report form to the amendment application by clicking 'Attach'. Then select the related Report Form from the list.

You can also submit a new Report Form from this amendment application by clicking 'New'. Follow the instructions for creating a new Report Form below.

ID	Name	Date Submitted	Status

IRB_00125797 - AM_Add Screen Recordings to the Interviews

Created: 12/19/2019 2:49 PM

AM_00036552
6. Documents and Attachments

PI: Kensaku Kawamoto

Submitted: 1/2/2020

Title: Design and Implementation of Scalable Decision Support and Shared Decision Making for Lung Cancer Screening

6. Documents and Attachments

Approved eProtocol Summary:

Name	Version	Date Created	Date Modified	Date Approved
ID00000007(0.01)	0.01	3/16/2023 2:52 PM	3/16/2023 2:52 PM	ID00000007

Print View: IRB Draft Protocol Summary

Updated eProtocol Summary:

Name	Version	Date Created	Date Modified	Date Approved

There are no items to display

Approved Consent Forms:

Name	Version	Date Created	Date Modified	Date Approved

There are no items to display

Updated Consent Forms:

Name	Version	Date Created	Date Modified	Date Approved

There are no items to display

Approved Parental Permission Forms:

Name	Version	Date Created	Date Modified	Date Approved

There are no items to display

Updated Parental Permission Forms:

Name	Version	Date Created	Date Modified	Date Approved

There are no items to display

Approved Assent Forms:

Name	Version	Date Created	Date Modified	Date Approved

There are no items to display

Updated Assent Forms:

Name	Version	Date Created	Date Modified	Date Approved

There are no items to display

Approved VA Consent Forms:

Name	Version	Date Created	Date Modified	Date Approved

There are no items to display

Updated VA Consent Forms:

Name	Version	Date Created	Date Modified	Date Approved

There are no items to display

Approved Surveys, etc.:

Name	Version	Date Created	Date Modified	Date Approved
Focus Group Guide (Patients) v20191016.docx(0.01)	0.01	10/17/2019 7:37 PM	10/17/2019 7:37 PM	11/6/2019 3:10 PM
Focus Group Guide (Providers) v20191016.docx(0.01)	0.01	10/17/2019 7:37 PM	10/17/2019 7:37 PM	11/6/2019 3:10 PM
Focus Group Guide (Staff) v20191016.docx(0.01)	0.01	10/17/2019 7:37 PM	10/17/2019 7:37 PM	11/6/2019 3:10 PM
Post-Implementation Interview Guide (Providers) v20191016.docx(0.01)	0.01	10/17/2019 7:37 PM	10/17/2019 7:37 PM	11/6/2019 3:10 PM
Post-Implementation Interview Guide (Staff) v20191016.docx(0.01)	0.01	10/17/2019 7:37 PM	10/17/2019 7:37 PM	11/6/2019 3:10 PM
Pre-Implementation Interview Guide (Implementation Stakeholders) v20191017.docx(0.01)	0.01	10/17/2019 7:37 PM	10/17/2019 7:37 PM	11/6/2019 3:10 PM
Pre-Implementation Interview Guide (Providers) v20191016.docx(0.01)	0.01	10/17/2019 7:37 PM	10/17/2019 7:37 PM	11/6/2019 3:10 PM
Pre-Implementation Interview Guide (Staff) v20191016.docx(0.01)	0.01	10/17/2019 7:37 PM	10/17/2019 7:37 PM	11/6/2019 3:10 PM
Survey (Patients) v20191018.docx(0.01)	0.01	10/18/2019 4:15 PM	10/18/2019 4:15 PM	11/6/2019 3:10 PM
Survey (Providers) v20220505.docx(0.01)	0.01	5/10/2022 3:33 PM	5/10/2022 3:33 PM	5/17/2022 11:16 AM

Updated Surveys, etc.:

Name	Version	Date Created	Date Modified	Date Approved

There are no items to display

Approved Company Protocol:

Name	Version	Date Created	Date Modified	Date Approved

There are no items to display

Updated Company Protocol:

Name	Version	Date Created	Date Modified	Date Approved

There are no items to display

Approved Investigational Brochure:

Name	Version	Date Created	Date Modified	Date Approved

There are no items to display

Updated Investigational Brochure:

Name	Version	Date Created	Date Modified	Date Approved

There are no items to display

Approved Grant Application:

Name	Version	Date Created	Date Modified	Date Approved
Grant Application(0.01)	0.01	9/6/2019 8:02 PM	9/6/2019 8:02 PM	11/6/2019 3:10 PM

Updated Grant Application:

Name	Version	Date Created	Date Modified	Date Approved

There are no items to display

Approved Literature/Cited References:

Name	Version	Date Created	Date Modified	Date Approved

There are no items to display

Updated Literature/Cited References:

Name	Version	Date Created	Date Modified	Date Approved

There are no items to display

Current PI Scholarly Record (CV/Resume):

Name	Version	Date Created	Date Modified	Date Approved
Kawamoto CV.pdf(0.05)	0.05	1/16/2014 12:24 PM	2/13/2024 9:09 AM	

Updated PI Scholarly Record (CV/Resume):

Name	Version	Date Created	Date Modified	Date Approved
Kawamoto CV.pdf(0.05)	0.05	1/16/2014 12:24 PM	2/13/2024 9:09 AM	

Current Faculty Sponsor Scholarly Record (CV/Resume):

Name	Version	Date Created	Date Modified	Date Approved

There are no items to display

Updated Faculty Sponsor Scholarly Record (CV/Resume):

Name	Version	Date Created	Date Modified	Date Approved

There are no items to display

Approved Other Stamped Documents:

Name	Version	Date Created	Date Modified	Date Approved

There are no items to display

Updated Other Stamped Documents:

Name	Version	Date Created	Date Modified	Date Approved

There are no items to display

Approved Recruitment Materials, Advertisements, etc.:

Name	Version	Date Created	Date Modified	Date Approved
Recruitment Email Sample - Interviews.docx(0.01)	0.01	10/18/2019 4:14 PM	10/18/2019 4:14 PM	11/6/2019 3:10 PM
Recruitment Email Sample - Surveys.docx(0.01)	0.01	10/18/2019 4:14 PM	10/18/2019 4:14 PM	11/6/2019 3:10 PM

Updated Recruitment Materials, Advertisements, etc.:

Name	Version	Date Created	Date Modified	Date Approved

There are no items to display

Approved Other Documents:

Name	Version	Date Created	Date Modified	Date Approved

There are no items to display

Updated Other Documents:

Name	Version	Date Created	Date Modified	Date Approved

There are no items to display

IRB_00125797 - AM_Add Screen Recordings to the Interviews **Created:** 12/19/2019 2:49 PM

AM_00036552
7. Finish

PI: Kensaku Kawamoto **Submitted:** 1/2/2020

Title: Design and Implementation of Scalable Decision Support and Shared Decision Making for Lung Cancer Screening

7. Instructions and Finish

1. To view errors in this application, select the "Validate" option at the top-left of the page. If you have errors on your application, you won't be able to submit it to the IRB.

Changes to the Update Study Application

2. Be sure to make all proposed changes to the Update Study portion of the application by selecting the "Update Study" button located on the left side of the amendment or continuing review workspace, which will be available once you select the "Finish" button at the top or bottom of this page.
3. To attach updated or new documents with this application, you may access the Documents and Attachments page in the Update Study application.
4. If you are proposing changes to any ancillary applications (i.e. RDRC-HUS or RGE), you must access these applications through the Update Study application on the Ancillary Applications page. All changes to ancillary applications must be approved by the corresponding committee prior to IRB approval of the amendment.

Submitting the Completed Amendment Application

5. Selecting the “Finish” button alone will NOT submit the application to the IRB. You MUST also select the "Submit" option on the workspace after you've selected the "Finish" button. Only the PI can submit the application to the IRB.
6. If your study has a faculty sponsor: Once the PI submits the application, it will be sent to the faculty sponsor for final approval. The IRB cannot review the study until the faculty sponsor submits the application to the IRB.

Electronic
RESEARCH INTEGRITY AND COMPLIANCE
THE UNIVERSITY OF UTAH

Date: Monday, February 26, 2024 8:40:07 AM

Print Close

IRB_00125797 - AM_Mitigating the impact of COVID19 pandemic

Created: 5/21/2020 3:43 PM

PI: Kensaku Kawamoto

Submitted: 6/5/2020

Title: Design and Implementation of Scalable Decision Support and Shared Decision Making for Lung Cancer Screening

AM_00038201

1. Amendment Type

1. Amendment Introduction

Brief Description of the Study: (This will populate from original application)

The purpose of this project is to increase appropriate low-dose computed tomography (LDCT) lung cancer screening through the development and wide dissemination of patient-centered clinical decision support (CDS) tools that (1) are integrated with the electronic health record (EHR) and clinical workflows, (2) prompt for shared decision making (SDM) when patients meet screening criteria, and (3) enable effective SDM using individually-tailored information on the potential benefits and harms of screening. The study will promote standard of care that is endorsed by the Centers for Medicare & Medicaid Services (CMS) and the US Preventive Services Task Force (USPSTF). This project is supported both operationally and by an Agency for Healthcare Research and Quality (AHRQ) R18 grant.

The EHR-based CDS tools are being developed by the University of Utah ReImagine EHR team, directed by Dr. Kensaku Kawamoto. Within University of Utah Health, Dr. Kawamoto is also Associate Chief Medical Information Officer and Director of the health system's Knowledge Management and Mobilization unit. Dr. Kawamoto's team receives operational funding from University of Utah Health to develop EHR-based solutions to improve patient care and the provider experience. Grant funding such as the current AHRQ R18 grant enable this operational team to allocate more resources to the optimal design, development, and implementation of these software tools, as well as to undertake more rigorous intervention design and evaluation procedures that would not be possible in the context of operational quality improvement (QI).

This project will leverage Decision Precision (https://share.lungdecisionprecision.com/), a validated Web-based tool for LDCT SDM developed at the Veterans Health Administration, as well as an initial version of Decision Precision+, an EHR-integrated version of the tool which can be accessed directly in the EHR and auto-populate relevant patient data in the tool instead of requiring manual data entry. An initial version of Decision Precision+ was developed and made accessible within the Epic EHR shortly after the AHRQ grant was submitted for operational QI purposes. However, due to feedback from physician leaders that the system needed to be streamlined for optimal use in busy primary care settings, the tool's availability was never advertised and no CDS prompts were introduced to encourage its use. Consequently, tool access has been minimal to date.

This study seeks to build upon our existing work in CDS and SDM to promote appropriate LDCT lung cancer screening as recommended by the USPSTF and CMS. Listed below are the three study aims, as well as the human subjects research associated with each aim.

Aim 1. System Design and Development

Design and develop a standards-based CDS tool for lung cancer screening SDM that is integrated with the EHR and can be effectively used in busy primary care settings (Decision Precision+); design and develop CDS tools for optimally integrating the tool into clinical workflows; and advance underlying standards and their adoption. Information for user-centered design and workflow assessments will be collected through cognitive work analysis interviews with clinicians at University of Utah Health. Human subjects research for this aim will consist of the following:

- Pre-Implementation Provider and Staff Interviews

Aim 2. Implementation Trial and Iterative System Refinement

Conduct a pragmatic implementation trial of Decision Precision+ and associated CDS tools within University of Utah Health. Make Decision Precision+ available to other institutions through app stores, and also share associated CDS tools. There will be no randomization, and the actual clinical trial will be preceded by a pilot implementation to ensure that the tools are useable and compatible with clinical workflows. The study population for the clinical trial will be primary care patients at University of Utah Health who are eligible for or potentially eligible for LDCT screening and the associated SDM, and the initial intervention will include availability of Decision Precision+ combined with CDS prompts to promote its appropriate use. The intervention will be enhanced based on feedback, with anticipated enhancements including prompting medical assistants to collect required smoking history information from potentially eligible patients, as well as prompting patients to consider LDCT screening and SDM via the personal health record. Human subjects research for this aim will consist of the following:

- Provider and Staff Participation in Pilot Implementation

- Patient Participation in Pilot Implementation

- Provider and Staff Participation in Clinical Trial

- Patient Participation in Clinical Trial

- Post-implementation Provider and Staff Interviews

Aim 3. Evaluation

Evaluate the impact of the CDS tool, including for adoption, clinical impact, and financial impact. We will conduct an interrupted time series (ITS) study of the implementation trial; focus groups and surveys with patients, staff, and providers; and evaluation of the resource costs associated with implementation. Human subjects research for this aim will consist of the following:

- Patient Outcomes Assessment

- Provider and Staff App Usage Assessment

- Provider and Staff Surveys

- Patient Surveys

- Provider and Staff Focus Groups

- Patient Focus Groups

- Stakeholder Interviews

1. **Name of Amendment:**
   Use a name that will make it easy to identify the contents of the amendment. You may use information such as the sponsor amendment number or an internal tracking number.
   Mitigating the impact of COVID19 pandemic

2. **Type of Amendment (check all that apply):**
   Changes to study procedures:

3. **Current Status of the Study:**
   Open for Enrollment

4. **Total Number of Participants Enrolled To Date**
   At Utah: 17
   All Centers: 17

IRB_00125797 - AM_Mitigating the impact of COVID19 pandemic **Created:** 5/21/2020 3:43 PM

AM_00038201
2. Description

PI: Kensaku Kawamoto **Submitted:** 6/5/2020

Title: Design and Implementation of Scalable Decision Support and Shared Decision Making for Lung Cancer Screening

2. Amendment Description

You have indicated that the following types of changes are being made:

Changes to study procedures:

1. **What changes are being made? List and number each change, grouping similar changes together.**

   1. We updated the statistical analysis section to mitigate the reduced number of outpatient visits during the COVID pandemic: this includes redefining the baseline phase to not overlap with the lock-down period and conducting propensity score modeling if the baseline patient distribution is significantly different from the intervention phases.

   2. We clarified that interview recording will be transcribed.

2. **Describe the reason for each of the changes described above. List and number the reasons according to the list above.**

   1. We are making this change to account for COVID-19.

   2. We had unintentionally omitted an explicit statement that interview recordings need to be transcribed prior to analysis.

3. **How does each change described above affect participants? List and number the effects according to the above list.**

   This changes will not affect participants.

4. **Will the modification(s), in the opinion of the local PI, increase or decrease the risk to participants?**
   Neither

   If the risk changes, provide justification:

5. **How will enrolled participants (current and past) be notified of this change?**
   Other:

   If Other, please explain:
   The update to the statistical analysis plan to account for COVID-19 does not need to be communicated because the trial has not yet started.

   With regard to transcribing interviews, the interview guide approved by the IRB previously explicitly included this information, as below. So no notification will be required:

   "We would like to record our discussion, audio and screen capture. The recordings will be reviewed by members of our study team and the audio recording may be sent for professional transcription. The recording and transcript will be identified by your participant ID, not your name."

6. **Which approved documents are affected by these changes?**
   There are no items to display

If other, please list:

7. **Which sections of the Update Study Application are affected by these changes?**
   4.6. Procedures

   4.9. Provide a summary of the statistical methods, data analysis, or data interpretation planned for this study. Factors for determining the proposed sample size (e.g., power) should be stated.

8. **Select all study locations that are affected by these changes.**

Prior Approved Study Controlled Locations

Site Name	Investigator Name	Covered Entity
☐ University of Utah	Kensaku Kawamoto	Study procedures will be conducted within a HIPAA Covered Entity at this site (HIPAA Privacy Rule applies)

New Sites

IRB_00125797 - AM_Mitigating the impact of COVID19 pandemic **Created:** 5/21/2020 3:43 PM

PI: Kensaku Kawamoto **Submitted:** 6/5/2020

Title: Design and Implementation of Scalable Decision Support and Shared Decision Making for Lung Cancer Screening

AM_00038201
3. Report Forms

3. Report Forms

1. **Is this amendment related to information that meets the IRB reporting policy for unanticipated problems and non-compliance?**
   ○ Yes ● **No**

If yes, a Report Form must also be submitted and then linked to this amendment. Link the related report form to the amendment application by clicking 'Attach'. Then select the related Report Form from the list.

You can also submit a new Report Form from this amendment application by clicking 'New'. Follow the instructions for creating a new Report Form below.

ID	Name	Date Submitted	Status

IRB_00125797 - AM_Mitigating the impact of COVID19 pandemic

Created: 5/21/2020 3:43 PM

PI: Kensaku Kawamoto

Submitted: 6/5/2020

Title: Design and Implementation of Scalable Decision Support and Shared Decision Making for Lung Cancer Screening

AM_00038201
6. Documents and Attachments

6. Documents and Attachments

Approved eProtocol Summary:

Name	Version	Date Created	Date Modified	Date Approved
ID00000007(0.01)	0.01	3/16/2023 2:52 PM	3/16/2023 2:52 PM	ID00000007

Print View: IRB Draft Protocol Summary

Updated eProtocol Summary:

Name	Version	Date Created	Date Modified	Date Approved

There are no items to display

Approved Consent Forms:

Name	Version	Date Created	Date Modified	Date Approved

There are no items to display

Updated Consent Forms:

Name	Version	Date Created	Date Modified	Date Approved

There are no items to display

Approved Parental Permission Forms:

Name	Version	Date Created	Date Modified	Date Approved

There are no items to display

Updated Parental Permission Forms:

Name	Version	Date Created	Date Modified	Date Approved

There are no items to display

Approved Assent Forms:

Name	Version	Date Created	Date Modified	Date Approved

There are no items to display

Updated Assent Forms:

Name	Version	Date Created	Date Modified	Date Approved

There are no items to display

Approved VA Consent Forms:

Name	Version	Date Created	Date Modified	Date Approved

There are no items to display

Updated VA Consent Forms:

Name	Version	Date Created	Date Modified	Date Approved

There are no items to display

Approved Surveys, etc.:

Name	Version	Date Created	Date Modified	Date Approved
Focus Group Guide (Patients) v20191016.docx(0.01)	0.01	10/17/2019 7:37 PM	10/17/2019 7:37 PM	11/6/2019 3:10 PM
Focus Group Guide (Providers) v20191016.docx(0.01)	0.01	10/17/2019 7:37 PM	10/17/2019 7:37 PM	11/6/2019 3:10 PM
Focus Group Guide (Staff) v20191016.docx(0.01)	0.01	10/17/2019 7:37 PM	10/17/2019 7:37 PM	11/6/2019 3:10 PM
Post-Implementation Interview Guide (Providers) v20191016.docx(0.01)	0.01	10/17/2019 7:37 PM	10/17/2019 7:37 PM	11/6/2019 3:10 PM
Post-Implementation Interview Guide (Staff) v20191016.docx(0.01)	0.01	10/17/2019 7:37 PM	10/17/2019 7:37 PM	11/6/2019 3:10 PM
Pre-Implementation Interview Guide (Implementation Stakeholders) v20191017.docx(0.01)	0.01	10/17/2019 7:37 PM	10/17/2019 7:37 PM	11/6/2019 3:10 PM
Pre-Implementation Interview Guide (Providers) v20191016.docx(0.01)	0.01	10/17/2019 7:37 PM	10/17/2019 7:37 PM	11/6/2019 3:10 PM
Pre-Implementation Interview Guide (Staff) v20191016.docx(0.01)	0.01	10/17/2019 7:37 PM	10/17/2019 7:37 PM	11/6/2019 3:10 PM
Survey (Patients) v20191018.docx(0.01)	0.01	10/18/2019 4:15 PM	10/18/2019 4:15 PM	11/6/2019 3:10 PM
Survey (Providers) v20220505.docx(0.01)	0.01	5/10/2022 3:33 PM	5/10/2022 3:33 PM	5/17/2022 11:16 AM

Updated Surveys, etc.:

Name	Version	Date Created	Date Modified	Date Approved

There are no items to display

Approved Company Protocol:

Name	Version	Date Created	Date Modified	Date Approved

There are no items to display

Updated Company Protocol:

Name	Version	Date Created	Date Modified	Date Approved

There are no items to display

Approved Investigational Brochure:

Name	Version	Date Created	Date Modified	Date Approved

There are no items to display

Updated Investigational Brochure:

Name	Version	Date Created	Date Modified	Date Approved

There are no items to display

Approved Grant Application:

Name	Version	Date Created	Date Modified	Date Approved
Grant Application(0.01)	0.01	9/6/2019 8:02 PM	9/6/2019 8:02 PM	11/6/2019 3:10 PM

Updated Grant Application:

Name	Version	Date Created	Date Modified	Date Approved

There are no items to display

Approved Literature/Cited References:

Name	Version	Date Created	Date Modified	Date Approved

There are no items to display

Updated Literature/Cited References:

Name	Version	Date Created	Date Modified	Date Approved

There are no items to display

Current PI Scholarly Record (CV/Resume):

Name	Version	Date Created	Date Modified	Date Approved
Kawamoto CV.pdf(0.05)	0.05	1/16/2014 12:24 PM	2/13/2024 9:09 AM	

Updated PI Scholarly Record (CV/Resume):

Name	Version	Date Created	Date Modified	Date Approved
Kawamoto CV.pdf(0.05)	0.05	1/16/2014 12:24 PM	2/13/2024 9:09 AM	

Current Faculty Sponsor Scholarly Record (CV/Resume):

Name	Version	Date Created	Date Modified	Date Approved

There are no items to display

Updated Faculty Sponsor Scholarly Record (CV/Resume):

Name	Version	Date Created	Date Modified	Date Approved

There are no items to display

Approved Other Stamped Documents:

Name	Version	Date Created	Date Modified	Date Approved

There are no items to display

Updated Other Stamped Documents:

Name	Version	Date Created	Date Modified	Date Approved

There are no items to display

Approved Recruitment Materials, Advertisements, etc.:

Name	Version	Date Created	Date Modified	Date Approved
Recruitment Email Sample - Interviews.docx(0.01)	0.01	10/18/2019 4:14 PM	10/18/2019 4:14 PM	11/6/2019 3:10 PM
Recruitment Email Sample - Surveys.docx(0.01)	0.01	10/18/2019 4:14 PM	10/18/2019 4:14 PM	11/6/2019 3:10 PM

Updated Recruitment Materials, Advertisements, etc.:

Name	Version	Date Created	Date Modified	Date Approved

There are no items to display

Approved Other Documents:

Name	Version	Date Created	Date Modified	Date Approved

There are no items to display

Updated Other Documents:

Name	Version	Date Created	Date Modified	Date Approved

There are no items to display

IRB_00125797 - AM_Mitigating the impact of COVID19 pandemic **Created:** 5/21/2020 3:43 PM

PI: Kensaku Kawamoto **Submitted:** 6/5/2020

Title: Design and Implementation of Scalable Decision Support and Shared Decision Making for Lung Cancer Screening

AM_00038201
7. Finish

7. Instructions and Finish

1. To view errors in this application, select the "Validate" option at the top-left of the page. If you have errors on your application, you won't be able to submit it to the IRB.

Changes to the Update Study Application

2. Be sure to make all proposed changes to the Update Study portion of the application by selecting the "Update Study" button located on the left side of the amendment or continuing review workspace, which will be available once you select the "Finish" button at the top or bottom of this page.
3. To attach updated or new documents with this application, you may access the Documents and Attachments page in the Update Study application.
4. If you are proposing changes to any ancillary applications (i.e. RDRC-HUS or RGE), you must access these applications through the Update Study application on the Ancillary Applications page. All changes to ancillary applications must be approved by the corresponding committee prior to IRB approval of the amendment.

Submitting the Completed Amendment Application

5. Selecting the “Finish” button alone will NOT submit the application to the IRB. You MUST also select the "Submit" option on the workspace after you've selected the "Finish" button. Only the PI can submit the application to the IRB.
6. If your study has a faculty sponsor: Once the PI submits the application, it will be sent to the faculty sponsor for final approval. The IRB cannot review the study until the faculty sponsor submits the application to the IRB.

Electronic
RESEARCH INTEGRITY AND COMPLIANCE
THE UNIVERSITY OF UTAH

Date: Monday, February 26, 2024 8:41:02 AM

Print Close

IRB_00125797 - AM_Changing Enrollment Goal and Quality Assurance Analysis

Created: 8/23/2021 10:04 AM

PI: Kensaku Kawamoto

Submitted: 8/24/2021

Title: Design and Implementation of Scalable Decision Support and Shared Decision Making for Lung Cancer Screening

AM_00042563

1. Amendment Type

1. Amendment Introduction

Brief Description of the Study: (This will populate from original application)

The purpose of this project is to increase appropriate low-dose computed tomography (LDCT) lung cancer screening through the development and wide dissemination of patient-centered clinical decision support (CDS) tools that (1) are integrated with the electronic health record (EHR) and clinical workflows, (2) prompt for shared decision making (SDM) when patients meet screening criteria, and (3) enable effective SDM using individually-tailored information on the potential benefits and harms of screening. The study will promote standard of care that is endorsed by the Centers for Medicare & Medicaid Services (CMS) and the US Preventive Services Task Force (USPSTF).The US Preventive Services Task Force (USPSTF) provided a grade B recommendation in 2013 that annual LDCT lung cancer screening be offered to patients 55 to 80 years old with a 30+ pack-year smoking history who are current smokers or quit in the last 15 years. In March 2021, the USPSTF updated its recommendation to lower the eligibility age to 50 and the smoking requirement to 20+ pack-years.

This project is supported both operationally and by an Agency for Healthcare Research and Quality (AHRQ) R18 grant.

The EHR-based CDS tools are being developed by the University of Utah ReImagine EHR team, directed by Dr. Kensaku Kawamoto. Within University of Utah Health, Dr. Kawamoto is also Associate Chief Medical Information Officer and Director of the health system's Knowledge Management and Mobilization unit. Dr. Kawamoto's team receives operational funding from University of Utah Health to develop EHR-based solutions to improve patient care and the provider experience. Grant funding such as the current AHRQ R18 grant enable this operational team to allocate more resources to the optimal design, development, and implementation of these software tools, as well as to undertake more rigorous intervention design and evaluation procedures that would not be possible in the context of operational quality improvement (QI).

This project will leverage Decision Precision (https://share.lungdecisionprecision.com/), a validated Web-based tool for LDCT SDM developed at the Veterans Health Administration, as well as an initial version of Decision Precision+, an EHR-integrated version of the tool which can be accessed directly in the EHR and auto-populate relevant patient data in the tool instead of requiring manual data entry. An initial version of Decision Precision+ was developed and made accessible within the Epic EHR shortly after the AHRQ grant was submitted for operational QI purposes. However, due to feedback from physician leaders that the system needed to be streamlined for optimal use in busy primary care settings, the tool's availability was never advertised and no CDS prompts were introduced to encourage its use. Consequently, tool access has been minimal to date.

This study seeks to build upon our existing work in CDS and SDM to promote appropriate LDCT lung cancer screening as recommended by the USPSTF and CMS. Listed below are the three study aims, as well as the human subjects research associated with each aim.

Aim 1. System Design and Development

Design and develop a standards-based CDS tool for lung cancer screening SDM that is integrated with the EHR and can be effectively used in busy primary care settings (Decision Precision+); design and develop CDS tools for optimally integrating the tool into clinical workflows; and advance underlying standards and their adoption. Information for user-centered design and

workflow assessments will be collected through cognitive work analysis interviews with clinicians at University of Utah Health. Human subjects research for this aim will consist of the following:

- Pre-Implementation Provider and Staff Interviews

Aim 2. Implementation Trial and Iterative System Refinement

Conduct a pragmatic implementation trial of Decision Precision+ and associated CDS tools within University of Utah Health. Make Decision Precision+ available to other institutions through app stores, and also share associated CDS tools. There will be no randomization, and the actual clinical trial will be preceded by a pilot implementation to ensure that the tools are useable and compatible with clinical workflows. The study population for the clinical trial will be primary care patients at University of Utah Health who are eligible for or potentially eligible for LDCT screening and the associated SDM, and the initial intervention will include availability of Decision Precision+ combined with CDS prompts to promote its appropriate use. The intervention will be enhanced based on feedback, with anticipated enhancements including prompting medical assistants to collect required smoking history information from potentially eligible patients, as well as prompting patients to consider LDCT screening and SDM via the personal health record. Human subjects research for this aim will consist of the following:

- Provider and Staff Participation in Pilot Implementation

- Patient Participation in Pilot Implementation

- Provider and Staff Participation in Clinical Trial

- Patient Participation in Clinical Trial

- Post-implementation Provider and Staff Interviews

Aim 3. Evaluation

Evaluate the impact of the CDS tool, including for adoption, clinical impact, and financial impact. We will conduct an interrupted time series (ITS) study of the implementation trial; focus groups and surveys with patients, staff, and providers; and evaluation of the resource costs associated with implementation. Human subjects research for this aim will consist of the following:

- Patient Outcomes Assessment

- Provider and Staff App Usage Assessment

- Provider and Staff Surveys

- Patient Surveys

- Provider and Staff Focus Groups

- Patient Focus Groups

- Stakeholder Interviews

1. **Name of Amendment:**
   Use a name that will make it easy to identify the contents of the amendment. You may use information such as the sponsor amendment number or an internal tracking number.
   Changing Enrollment Goal and Quality Assurance Analysis

2. **Type of Amendment (check all that apply):**
   Changes to study design elements:

   Changes to study procedures:

3. **Current Status of the Study:**
   Open for Enrollment

4. **Total Number of Participants Enrolled To Date**
   At Utah: 4736
   All Centers: 4736

IRB_00125797 - AM_Changing Enrollment Goal and Quality Assurance Analysis

Created: 8/23/2021 10:04 AM

AM_00042563
2. Description

PI: Kensaku Kawamoto

Submitted: 8/24/2021

Title: Design and Implementation of Scalable Decision Support and Shared Decision Making for Lung Cancer Screening

2. Amendment Description

You have indicated that the following types of changes are being made:

Changes to study design elements:

Changes to study procedures:

1. **What changes are being made? List and number each change, grouping similar changes together.**

   1. Changing the number of participants (enrollment goal): Patient enrollment goal is changed from 12,000 to 21,000.

   2. We would like to formally study potential impact of data errors in smoking history data on patient eligibility for lung cancer screening. To formally study such impact, we will quantify inaccuracies in smoking data, predict pack-years and years quit based on corrected data and estimate how many patients our lung cancer screening program is not reaching because of the inaccurate smoking history data.

2. **Describe the reason for each of the changes described above. List and number the reasons according to the list above.**

   1. Changing the number of participants (enrollment goal): The US Preventive Services Task Force (USPSTF) provided a grade B recommendation in 2013 that annual LDCT lung cancer screening be offered to patients 55 to 80 years old with a 30+ pack-year smoking history who are current smokers or quit in the last 15 years. In March 2021, the USPSTF updated its recommendation to lower the eligibility age to 50 and the smoking requirement to 20+ pack-years. Due to this change in clinical guidelines, our enrollment goal is changing to include 50-55 year old patients who were previously omitted. Up to 200,000 50-80 patients will be assessed to determine their smoking status and history of lung cancer. Out of those, up to 70,000 (35%) are expected to be current or former smokers. Of those, 42,000 (60%) are expected to have sufficient smoking history to determine their eligibility for lung cancer screening. Of those, 21,000 (50%) might be potentially eligible for lung cancer screening. Thus, the patient enrollment goal is changed from 12,000 to 21,000.

   2. Changing data analysis procedures: Since the beginning of this study we have been conducting rigorous data quality assurance. Since quality of smoking history data in the EHR is poor, we would like to formally study potential impact of data errors in smoking history data on patient eligibility for lung cancer screening.

3. **How does each change described above affect participants? List and number the effects according to the above list.**

   1. The expansion of the eligible participant population will not affect currently enrolled participants.

   2. The formal quality assurance study will have no direct effect on participants, but we will make an effort to disseminate this information through a peer reviewed publication, which may eventually results in better smoking history data collection practices.

4. **Will the modification(s), in the opinion of the local PI, increase or decrease the risk to participants?**
Neither

If the risk changes, provide justification:

5. **How will enrolled participants (current and past) be notified of this change?**
Participants will not be notified

If Other, please explain:

6. **Which approved documents are affected by these changes?**
There are no items to display

If other, please list:

7. **Which sections of the Update Study Application are affected by these changes?**
1. Study Introduction
1.6 Study Purposes and Objectives

4. Study Information
4.4.b Describe the recruitment/participant identification process in detail
4.6 Procedures
4.9. Provide a summary of the statistical methods, data analysis, or data interpretation planned for this study. Factors for determining the proposed sample size (e.g., power) should be stated.

6. Risks and Benefits
6.1 Describe the reasonable foreseeable risks or discomforts to the participants
6.2 Describe the potential benefits to society AND to participants

8. **Select all study locations that are affected by these changes.**

Prior Approved Study Controlled Locations

Site Name	Investigator Name	Covered Entity
☐ University of Utah	Kensaku Kawamoto	Study procedures will be conducted within a HIPAA Covered Entity at this site (HIPAA Privacy Rule applies)

New Sites

IRB_00125797 - AM_Changing Enrollment Goal and Quality Assurance Analysis

Created: 8/23/2021 10:04 AM

AM_00042563
3. Report Forms

PI: Kensaku Kawamoto

Submitted: 8/24/2021

Title: Design and Implementation of Scalable Decision Support and Shared Decision Making for Lung Cancer Screening

3. Report Forms

1. **Is this amendment related to information that meets the IRB reporting policy for unanticipated problems and non-compliance?**

   ○ Yes ● **No**

If yes, a Report Form must also be submitted and then linked to this amendment. Link the related report form to the amendment application by clicking 'Attach'. Then select the related Report Form from the list.

You can also submit a new Report Form from this amendment application by clicking 'New'. Follow the instructions for creating a new Report Form below.

ID	Name	Date Submitted	Status

IRB_00125797 - AM_Changing Enrollment Goal and Quality Assurance Analysis

Created: 8/23/2021 10:04 AM

PI: Kensaku Kawamoto

Submitted: 8/24/2021

Title: Design and Implementation of Scalable Decision Support and Shared Decision Making for Lung Cancer Screening

AM_00042563

6. Documents and Attachments

6. Documents and Attachments

Approved eProtocol Summary:

Name	Version	Date Created	Date Modified	Date Approved
ID00000007(0.01)	0.01	3/16/2023 2:52 PM	3/16/2023 2:52 PM	ID00000007

Print View: IRB Draft Protocol Summary

Updated eProtocol Summary:

Name	Version	Date Created	Date Modified	Date Approved

There are no items to display

Approved Consent Forms:

Name	Version	Date Created	Date Modified	Date Approved

There are no items to display

Updated Consent Forms:

Name	Version	Date Created	Date Modified	Date Approved

There are no items to display

Approved Parental Permission Forms:

Name	Version	Date Created	Date Modified	Date Approved

There are no items to display

Updated Parental Permission Forms:

Name	Version	Date Created	Date Modified	Date Approved

There are no items to display

Approved Assent Forms:

Name	Version	Date Created	Date Modified	Date Approved

There are no items to display

Updated Assent Forms:

Name	Version	Date Created	Date Modified	Date Approved

There are no items to display

Approved VA Consent Forms:

Name	Version	Date Created	Date Modified	Date Approved

There are no items to display

Updated VA Consent Forms:

Name	Version	Date Created	Date Modified	Date Approved

There are no items to display

Approved Surveys, etc.:

Name	Version	Date Created	Date Modified	Date Approved
Focus Group Guide (Patients) v20191016.docx(0.01)	0.01	10/17/2019 7:37 PM	10/17/2019 7:37 PM	11/6/2019 3:10 PM
Focus Group Guide (Providers) v20191016.docx(0.01)	0.01	10/17/2019 7:37 PM	10/17/2019 7:37 PM	11/6/2019 3:10 PM
Focus Group Guide (Staff) v20191016.docx(0.01)	0.01	10/17/2019 7:37 PM	10/17/2019 7:37 PM	11/6/2019 3:10 PM
Post-Implementation Interview Guide (Providers) v20191016.docx(0.01)	0.01	10/17/2019 7:37 PM	10/17/2019 7:37 PM	11/6/2019 3:10 PM
Post-Implementation Interview Guide (Staff) v20191016.docx(0.01)	0.01	10/17/2019 7:37 PM	10/17/2019 7:37 PM	11/6/2019 3:10 PM
Pre-Implementation Interview Guide (Implementation Stakeholders) v20191017.docx(0.01)	0.01	10/17/2019 7:37 PM	10/17/2019 7:37 PM	11/6/2019 3:10 PM
Pre-Implementation Interview Guide (Providers) v20191016.docx(0.01)	0.01	10/17/2019 7:37 PM	10/17/2019 7:37 PM	11/6/2019 3:10 PM
Pre-Implementation Interview Guide (Staff) v20191016.docx(0.01)	0.01	10/17/2019 7:37 PM	10/17/2019 7:37 PM	11/6/2019 3:10 PM
Survey (Patients) v20191018.docx(0.01)	0.01	10/18/2019 4:15 PM	10/18/2019 4:15 PM	11/6/2019 3:10 PM
Survey (Providers) v20220505.docx(0.01)	0.01	5/10/2022 3:33 PM	5/10/2022 3:33 PM	5/17/2022 11:16 AM

Updated Surveys, etc.:

Name	Version	Date Created	Date Modified	Date Approved

There are no items to display

Approved Company Protocol:

Name	Version	Date Created	Date Modified	Date Approved

There are no items to display

Updated Company Protocol:

Name	Version	Date Created	Date Modified	Date Approved

There are no items to display

Approved Investigational Brochure:

Name	Version	Date Created	Date Modified	Date Approved

There are no items to display

Updated Investigational Brochure:

Name	Version	Date Created	Date Modified	Date Approved

There are no items to display

Approved Grant Application:

Name	Version	Date Created	Date Modified	Date Approved
Grant Application(0.01)	0.01	9/6/2019 8:02 PM	9/6/2019 8:02 PM	11/6/2019 3:10 PM

Updated Grant Application:

Name	Version	Date Created	Date Modified	Date Approved

There are no items to display

Approved Literature/Cited References:

Name	Version	Date Created	Date Modified	Date Approved

There are no items to display

Updated Literature/Cited References:

Name	Version	Date Created	Date Modified	Date Approved

There are no items to display

Current PI Scholarly Record (CV/Resume):

Name	Version	Date Created	Date Modified	Date Approved
Kawamoto CV.pdf(0.05)	0.05	1/16/2014 12:24 PM	2/13/2024 9:09 AM	

Updated PI Scholarly Record (CV/Resume):

Name	Version	Date Created	Date Modified	Date Approved
Kawamoto CV.pdf(0.05)	0.05	1/16/2014 12:24 PM	2/13/2024 9:09 AM	

Current Faculty Sponsor Scholarly Record (CV/Resume):

Name	Version	Date Created	Date Modified	Date Approved

There are no items to display

Updated Faculty Sponsor Scholarly Record (CV/Resume):

Name	Version	Date Created	Date Modified	Date Approved

There are no items to display

Approved Other Stamped Documents:

Name	Version	Date Created	Date Modified	Date Approved

There are no items to display

Updated Other Stamped Documents:

Name	Version	Date Created	Date Modified	Date Approved

There are no items to display

Approved Recruitment Materials, Advertisements, etc.:

Name	Version	Date Created	Date Modified	Date Approved
Recruitment Email Sample - Interviews.docx(0.01)	0.01	10/18/2019 4:14 PM	10/18/2019 4:14 PM	11/6/2019 3:10 PM
Recruitment Email Sample - Surveys.docx(0.01)	0.01	10/18/2019 4:14 PM	10/18/2019 4:14 PM	11/6/2019 3:10 PM

Updated Recruitment Materials, Advertisements, etc.:

Name	Version	Date Created	Date Modified	Date Approved

There are no items to display

Approved Other Documents:

Name	Version	Date Created	Date Modified	Date Approved

There are no items to display

Updated Other Documents:

Name	Version	Date Created	Date Modified	Date Approved

There are no items to display

IRB_00125797 - AM_Changing Enrollment Goal and Quality Assurance Analysis **Created:** 8/23/2021 10:04 AM

AM_00042563
7. Finish

PI: Kensaku Kawamoto **Submitted:** 8/24/2021

Title: Design and Implementation of Scalable Decision Support and Shared Decision Making for Lung Cancer Screening

7. Instructions and Finish

1. To view errors in this application, select the "Validate" option at the top-left of the page. If you have errors on your application, you won't be able to submit it to the IRB.

Changes to the Update Study Application

2. Be sure to make all proposed changes to the Update Study portion of the application by selecting the "Update Study" button located on the left side of the amendment or continuing review workspace, which will be available once you select the "Finish" button at the top or bottom of this page.

3. To attach updated or new documents with this application, you may access the Documents and Attachments page in the Update Study application.

4. If you are proposing changes to any ancillary applications (i.e. RDRC-HUS or RGE), you must access these applications through the Update Study application on the Ancillary Applications page. All changes to ancillary applications must be approved by the corresponding committee prior to IRB approval of the amendment.

Submitting the Completed Amendment Application

5. Selecting the “Finish” button alone will NOT submit the application to the IRB. You MUST also select the "Submit" option on the workspace after you've selected the "Finish" button. Only the PI can submit the application to the IRB.

6. If your study has a faculty sponsor: Once the PI submits the application, it will be sent to the faculty sponsor for final approval. The IRB cannot review the study until the faculty sponsor submits the application to the IRB.

Electronic
RESEARCH INTEGRITY AND COMPLIANCE
THE UNIVERSITY OF UTAH

Date: Monday, February 26, 2024 8:41:45 AM

Print Close

IRB_00125797 - AM_Minor updates to provider survey questions

Created: 5/10/2022 3:28 PM

PI: Kensaku Kawamoto

Submitted: 5/10/2022

Title: Design and Implementation of Scalable Decision Support and Shared Decision Making for Lung Cancer Screening

AM_00044863

1. Amendment Type

1. Amendment Introduction

Brief Description of the Study: (This will populate from original application)

The purpose of this project is to increase appropriate low-dose computed tomography (LDCT) lung cancer screening through the development and wide dissemination of patient-centered clinical decision support (CDS) tools that (1) are integrated with the electronic health record (EHR) and clinical workflows, (2) prompt for shared decision making (SDM) when patients meet screening criteria, and (3) enable effective SDM using individually-tailored information on the potential benefits and harms of screening. The study will promote standard of care that is endorsed by the Centers for Medicare & Medicaid Services (CMS) and the US Preventive Services Task Force (USPSTF).The US Preventive Services Task Force (USPSTF) provided a grade B recommendation in 2013 that annual LDCT lung cancer screening be offered to patients 55 to 80 years old with a 30+ pack-year smoking history who are current smokers or quit in the last 15 years. In March 2021, the USPSTF updated its recommendation to lower the eligibility age to 50 and the smoking requirement to 20+ pack-years.

This project is supported both operationally and by an Agency for Healthcare Research and Quality (AHRQ) R18 grant.

The EHR-based CDS tools are being developed by the University of Utah ReImagine EHR team, directed by Dr. Kensaku Kawamoto. Within University of Utah Health, Dr. Kawamoto is also Associate Chief Medical Information Officer and Director of the health system's Knowledge Management and Mobilization unit. Dr. Kawamoto's team receives operational funding from University of Utah Health to develop EHR-based solutions to improve patient care and the provider experience. Grant funding such as the current AHRQ R18 grant enable this operational team to allocate more resources to the optimal design, development, and implementation of these software tools, as well as to undertake more rigorous intervention design and evaluation procedures that would not be possible in the context of operational quality improvement (QI).

This project will leverage Decision Precision (https://share.lungdecisionprecision.com/), a validated Web-based tool for LDCT SDM developed at the Veterans Health Administration, as well as an initial version of Decision Precision+, an EHR-integrated version of the tool which can be accessed directly in the EHR and auto-populate relevant patient data in the tool instead of requiring manual data entry. An initial version of Decision Precision+ was developed and made accessible within the Epic EHR shortly after the AHRQ grant was submitted for operational QI purposes. However, due to feedback from physician leaders that the system needed to be streamlined for optimal use in busy primary care settings, the tool's availability was never advertised and no CDS prompts were introduced to encourage its use. Consequently, tool access has been minimal to date.

This study seeks to build upon our existing work in CDS and SDM to promote appropriate LDCT lung cancer screening as recommended by the USPSTF and CMS. Listed below are the three study aims, as well as the human subjects research associated with each aim.

Aim 1. System Design and Development

Design and develop a standards-based CDS tool for lung cancer screening SDM that is integrated with the EHR and can be effectively used in busy primary care settings (Decision Precision+); design and develop CDS tools for optimally integrating the tool into clinical workflows; and advance underlying standards and their adoption. Information for user-centered design and

workflow assessments will be collected through cognitive work analysis interviews with clinicians at University of Utah Health. Human subjects research for this aim will consist of the following:

- Pre-Implementation Provider and Staff Interviews

Aim 2. Implementation Trial and Iterative System Refinement

Conduct a pragmatic implementation trial of Decision Precision+ and associated CDS tools within University of Utah Health. Make Decision Precision+ available to other institutions through app stores, and also share associated CDS tools. There will be no randomization, and the actual clinical trial will be preceded by a pilot implementation to ensure that the tools are useable and compatible with clinical workflows. The study population for the clinical trial will be primary care patients at University of Utah Health who are eligible for or potentially eligible for LDCT screening and the associated SDM, and the initial intervention will include availability of Decision Precision+ combined with CDS prompts to promote its appropriate use. The intervention will be enhanced based on feedback, with anticipated enhancements including prompting medical assistants to collect required smoking history information from potentially eligible patients, as well as prompting patients to consider LDCT screening and SDM via the personal health record. Human subjects research for this aim will consist of the following:

- Provider and Staff Participation in Pilot Implementation

- Patient Participation in Pilot Implementation

- Provider and Staff Participation in Clinical Trial

- Patient Participation in Clinical Trial

- Post-implementation Provider and Staff Interviews

Aim 3. Evaluation

Evaluate the impact of the CDS tool, including for adoption, clinical impact, and financial impact. We will conduct an interrupted time series (ITS) study of the implementation trial; focus groups and surveys with patients, staff, and providers; and evaluation of the resource costs associated with implementation. Human subjects research for this aim will consist of the following:

- Patient Outcomes Assessment

- Provider and Staff App Usage Assessment

- Provider and Staff Surveys

- Patient Surveys

- Provider and Staff Focus Groups

- Patient Focus Groups

- Stakeholder Interviews

1. **Name of Amendment:**
   Use a name that will make it easy to identify the contents of the amendment. You may use information such as the sponsor amendment number or an internal tracking number.
   Minor updates to provider survey questions

2. **Type of Amendment (check all that apply):**
   Other changes

3. **Current Status of the Study:**
Open for Enrollment

4. **Total Number of Participants Enrolled To Date**
At Utah: 1777
All Centers: 1777

IRB_00125797 - AM_Minor updates to provider survey questions **Created:** 5/10/2022 3:28 PM

PI: Kensaku Kawamoto **Submitted:** 5/10/2022

Title: Design and Implementation of Scalable Decision Support and Shared Decision Making for Lung Cancer Screening

AM_00044863
2. Description

2. Amendment Description

You have indicated that the following types of changes are being made:

Other changes

1. **What changes are being made? List and number each change, grouping similar changes together.**

   1. Minor updates to provider survey questions to collect demographic data while keeping overall survey length/# of questions the same.

2. **Describe the reason for each of the changes described above. List and number the reasons according to the list above.**

   1. The minor update to provider survey questions is needed to collect demographic data for reporting purposes.

3. **How does each change described above affect participants? List and number the effects according to the above list.**

   1. The minor update to provider survey questions will have no effect on participants.

4. **Will the modification(s), in the opinion of the local PI, increase or decrease the risk to participants?**
   Neither

   If the risk changes, provide justification:

5. **How will enrolled participants (current and past) be notified of this change?**

   Participants will not be notified

   If Other, please explain:

6. **Which approved documents are affected by these changes?**

   Surveys, Questionnaires, Interview Scripts, etc.

   If other, please list:

7. **Which sections of the Update Study Application are affected by these changes?**
   The provider survey attachment is updated.

8. **Select all study locations that are affected by these changes.**

   Prior Approved Study Controlled Locations

Site Name	Investigator Name	Covered Entity
☐ University of Utah	Kensaku Kawamoto	Study procedures will be conducted within a HIPAA Covered Entity at this site (HIPAA Privacy Rule applies)

New Sites

IRB_00125797 - AM_Minor updates to provider survey questions

Created: 5/10/2022 3:28 PM

PI: Kensaku Kawamoto

Submitted: 5/10/2022

Title: Design and Implementation of Scalable Decision Support and Shared Decision Making for Lung Cancer Screening

AM_00044863
3. Report Forms

3. Report Forms

1. **Is this amendment related to information that meets the IRB reporting policy for unanticipated problems and non-compliance?**
   ◯ Yes ● **No**

If yes, a Report Form must also be submitted and then linked to this amendment. Link the related report form to the amendment application by clicking ‘Attach’. Then select the related Report Form from the list.

You can also submit a new Report Form from this amendment application by clicking ‘New’. Follow the instructions for creating a new Report Form below.

ID	Name	Date Submitted	Status

IRB_00125797 - AM_Minor updates to provider survey questions

Created: 5/10/2022 3:28 PM

AM_00044863
6. Documents and Attachments

PI: Kensaku Kawamoto

Submitted: 5/10/2022

Title: Design and Implementation of Scalable Decision Support and Shared Decision Making for Lung Cancer Screening

6. Documents and Attachments

Approved eProtocol Summary:

Name	Version	Date Created	Date Modified	Date Approved
ID00000007(0.01)	0.01	3/16/2023 2:52 PM	3/16/2023 2:52 PM	ID00000007

Print View: IRB Draft Protocol Summary

Updated eProtocol Summary:

Name	Version	Date Created	Date Modified	Date Approved

There are no items to display

Approved Consent Forms:

Name	Version	Date Created	Date Modified	Date Approved

There are no items to display

Updated Consent Forms:

Name	Version	Date Created	Date Modified	Date Approved

There are no items to display

Approved Parental Permission Forms:

Name	Version	Date Created	Date Modified	Date Approved

There are no items to display

Updated Parental Permission Forms:

Name	Version	Date Created	Date Modified	Date Approved

There are no items to display

Approved Assent Forms:

Name	Version	Date Created	Date Modified	Date Approved

There are no items to display

Updated Assent Forms:

Name	Version	Date Created	Date Modified	Date Approved

There are no items to display

Approved VA Consent Forms:

Name	Version	Date Created	Date Modified	Date Approved

There are no items to display

Updated VA Consent Forms:

Name	Version	Date Created	Date Modified	Date Approved

There are no items to display

Approved Surveys, etc.:

Name	Version	Date Created	Date Modified	Date Approved
Focus Group Guide (Patients) v20191016.docx(0.01)	0.01	10/17/2019 7:37 PM	10/17/2019 7:37 PM	11/6/2019 3:10 PM
Focus Group Guide (Providers) v20191016.docx(0.01)	0.01	10/17/2019 7:37 PM	10/17/2019 7:37 PM	11/6/2019 3:10 PM
Focus Group Guide (Staff) v20191016.docx(0.01)	0.01	10/17/2019 7:37 PM	10/17/2019 7:37 PM	11/6/2019 3:10 PM
Post-Implementation Interview Guide (Providers) v20191016.docx(0.01)	0.01	10/17/2019 7:37 PM	10/17/2019 7:37 PM	11/6/2019 3:10 PM
Post-Implementation Interview Guide (Staff) v20191016.docx(0.01)	0.01	10/17/2019 7:37 PM	10/17/2019 7:37 PM	11/6/2019 3:10 PM
Pre-Implementation Interview Guide (Implementation Stakeholders) v20191017.docx(0.01)	0.01	10/17/2019 7:37 PM	10/17/2019 7:37 PM	11/6/2019 3:10 PM
Pre-Implementation Interview Guide (Providers) v20191016.docx(0.01)	0.01	10/17/2019 7:37 PM	10/17/2019 7:37 PM	11/6/2019 3:10 PM
Pre-Implementation Interview Guide (Staff) v20191016.docx(0.01)	0.01	10/17/2019 7:37 PM	10/17/2019 7:37 PM	11/6/2019 3:10 PM
Survey (Patients) v20191018.docx(0.01)	0.01	10/18/2019 4:15 PM	10/18/2019 4:15 PM	11/6/2019 3:10 PM
Survey (Providers) v20220505.docx(0.01)	0.01	5/10/2022 3:33 PM	5/10/2022 3:33 PM	5/17/2022 11:16 AM

Updated Surveys, etc.:

Name	Version	Date Created	Date Modified	Date Approved

There are no items to display

Approved Company Protocol:

Name	Version	Date Created	Date Modified	Date Approved

There are no items to display

Updated Company Protocol:

Name	Version	Date Created	Date Modified	Date Approved

There are no items to display

Approved Investigational Brochure:

Name	Version	Date Created	Date Modified	Date Approved

There are no items to display

Updated Investigational Brochure:

Name	Version	Date Created	Date Modified	Date Approved

There are no items to display

Approved Grant Application:

Name	Version	Date Created	Date Modified	Date Approved
Grant Application(0.01)	0.01	9/6/2019 8:02 PM	9/6/2019 8:02 PM	11/6/2019 3:10 PM

Updated Grant Application:

Name	Version	Date Created	Date Modified	Date Approved

There are no items to display

Approved Literature/Cited References:

Name	Version	Date Created	Date Modified	Date Approved

There are no items to display

Updated Literature/Cited References:

Name	Version	Date Created	Date Modified	Date Approved

There are no items to display

Current PI Scholarly Record (CV/Resume):

Name	Version	Date Created	Date Modified	Date Approved
Kawamoto CV.pdf(0.05)	0.05	1/16/2014 12:24 PM	2/13/2024 9:09 AM	

Updated PI Scholarly Record (CV/Resume):

Name	Version	Date Created	Date Modified	Date Approved
Kawamoto CV.pdf(0.05)	0.05	1/16/2014 12:24 PM	2/13/2024 9:09 AM	

Current Faculty Sponsor Scholarly Record (CV/Resume):

Name	Version	Date Created	Date Modified	Date Approved

There are no items to display

Updated Faculty Sponsor Scholarly Record (CV/Resume):

Name	Version	Date Created	Date Modified	Date Approved

There are no items to display

Approved Other Stamped Documents:

Name	Version	Date Created	Date Modified	Date Approved

There are no items to display

Updated Other Stamped Documents:

Name	Version	Date Created	Date Modified	Date Approved
There are no items to display				

Approved Recruitment Materials, Advertisements, etc.:

Name	Version	Date Created	Date Modified	Date Approved
Recruitment Email Sample - Interviews.docx(0.01)	0.01	10/18/2019 4:14 PM	10/18/2019 4:14 PM	11/6/2019 3:10 PM
Recruitment Email Sample - Surveys.docx(0.01)	0.01	10/18/2019 4:14 PM	10/18/2019 4:14 PM	11/6/2019 3:10 PM

Updated Recruitment Materials, Advertisements, etc.:

Name	Version	Date Created	Date Modified	Date Approved
There are no items to display				

Approved Other Documents:

Name	Version	Date Created	Date Modified	Date Approved
There are no items to display				

Updated Other Documents:

Name	Version	Date Created	Date Modified	Date Approved
There are no items to display				

IRB_00125797 - AM_Minor updates to provider survey questions

Created: 5/10/2022 3:28 PM

AM_00044863
7. Finish

PI: Kensaku Kawamoto

Submitted: 5/10/2022

Title: Design and Implementation of Scalable Decision Support and Shared Decision Making for Lung Cancer Screening

7. Instructions and Finish

1. To view errors in this application, select the "Validate" option at the top-left of the page. If you have errors on your application, you won't be able to submit it to the IRB.

Changes to the Update Study Application

2. Be sure to make all proposed changes to the Update Study portion of the application by selecting the "Update Study" button located on the left side of the amendment or continuing review workspace, which will be available once you select the "Finish" button at the top or bottom of this page.

3. To attach updated or new documents with this application, you may access the Documents and Attachments page in the Update Study application.

4. If you are proposing changes to any ancillary applications (i.e. RDRC-HUS or RGE), you must access these applications through the Update Study application on the Ancillary Applications page. All changes to ancillary applications must be approved by the corresponding committee prior to IRB approval of the amendment.

Submitting the Completed Amendment Application

5. Selecting the “Finish” button alone will NOT submit the application to the IRB. You MUST also select the "Submit" option on the workspace after you've selected the "Finish" button. Only the PI can submit the application to the IRB.

6. If your study has a faculty sponsor: Once the PI submits the application, it will be sent to the faculty sponsor for final approval. The IRB cannot review the study until the faculty sponsor submits the application to the IRB.
