# Supplementary material for: Lung Cancer Screening Before and After a Multifaceted Electronic Health Record Intervention: A Nonrandomized Controlled Trial
Source: JAMA Netw Open. 2024 Jun 7;7(6):e2415383. doi: 10.1001/jamanetworkopen.2024.15383 (PMC11161845; doi:10.1001/jamanetworkopen.2024.15383)
Supplement: Supplement 2. — eMethods. Details of Clinician-Facing Period 1 Intervention eFigure 1. Study Flowchart eTable 1. Patient Inclusion and Exclusion Criteria eTable 2. Overall Patient Population Demographics eFigure 2. Covariate Balancing Propensity Score Model and Improvement in Covariates Balance for Baseline and Intervention Periods 1 and 2 [file jamanetwopen-e2415383-s002.pdf]

Supplemental Online Content

Kukhareva PV, Li H, Caverly TJ, et al. Lung cancer screening before and after a multifaceted electronic health record intervention: a nonrandomized controlled trial. *JAMA Netw Open*. 2024;7(6):e2415383. doi:10.1001/jamanetworkopen.2024.15383

eMethods. Details of Clinician-Facing Period 1 Intervention

eFigure 1. Study Flowchart

eTable 1. Patient Inclusion and Exclusion Criteria

eTable 2. Overall Patient Population Demographics

eFigure 2. Covariate Balancing Propensity Score Model and Improvement in Covariates Balance for Baseline and Intervention Periods 1 and 2

This supplemental material has been provided by the authors to give readers additional information about their work.

© 2024 Kukhareva PV et al. *JAMA Network Open.*

eMethods. Details of Clinician-Facing Period 1 Intervention

The shared decision making (SDM) tool was initially designed as a web-based tool within the Veterans Administration (VA) system to support SDM. This original tool worked well during dedicated, centralized lung cancer screening (LCS) SDM sessions in the VA. However, when evaluated in VA primary care and reviewed by University of Utah Health (UUH) clinicians, a more streamlined approach was needed due to time constraints. The tool was therefore adapted, retaining the most important SDM elements on the main page and moving the additional content to supplemental tabs. The tool was integrated with the electronic health record (EHR) using the Substitutable Medical Applications Reusable Technologies on Fast Healthcare Interoperability Resources (SMART on FHIR) interoperability framework. Time-saving features included generating Centers for Medicare and Medicaid Services (CMS)-required documentation, ordering low-dose computed tomography (LDCT) scans, and providing personalized conversation scripts. The EHR-integrated SDM tool presents the individual patient risk of developing lung cancer which is an important component of LCS decision making. Clinician-facing reminders were shown during every outpatient visit until they were addressed. The LDCT ordering screen always showed narrative guidance on the CMS SDM requirements; in addition, if the patient did not have a prior LDCT order, the provider was prompted to acknowledge that CMS required documentation of SDM prior to the baseline screen, and that the SDM tool should be used. The SDM tool had to be opened separately by searching for and opening the “Lung Cancer Screening” tool.

© 2024 Kukhareva PV et al. *JAMA Network Open.*

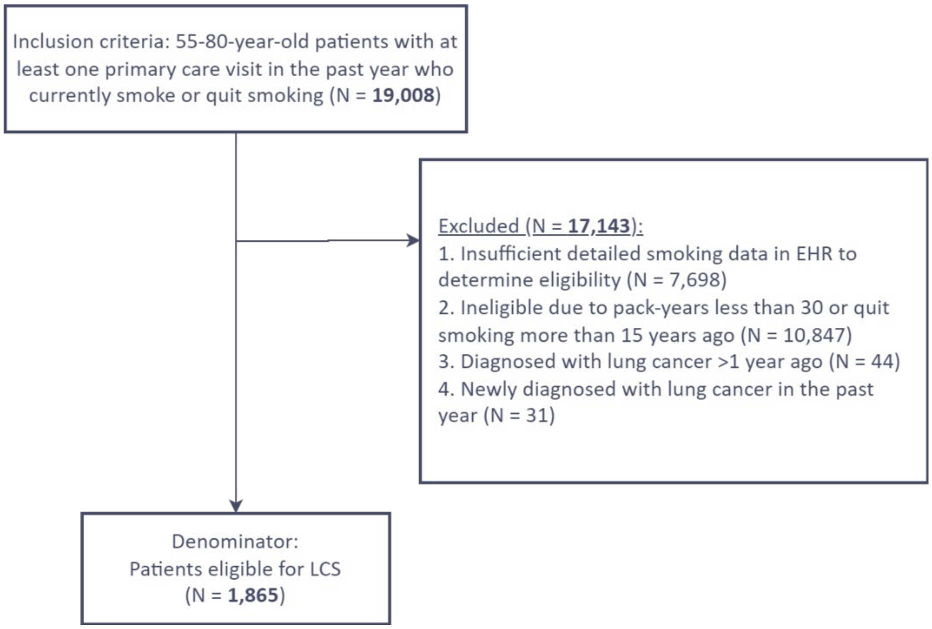

e-Figure S1. CONSORT diagram

© 2024 Kukhareva PV et al. *JAMA Network Open.*

e-Table S1. Patient inclusion and exclusion criteria

Inclusion and Exclusion Criteria	Baseline Period	Intervention Period 1	Intervention Period 2	Overall
Inclusion criteria: 55-80 year old patients with at least one primary care visit in the past year who currently smoke or quit smoking	12797	13631	14371	19008
Excluded: Insufficient detailed smoking data in EHR to determine eligibility	4622	4665	4866	7698
- Current smoker with missing pack-years	1501	1462	1486	2689
- Former smoker with missing pack-years and quit date	1661	1738	1874	3007
- Former smoker with missing pack-years and quit date < 15 years ago	1064	1109	1170	1845
- Former smoker with pack-years 30+ and missing quit date	374	353	334	578
- Unknown whether current or former smoker	22	3	2	26
Excluded: Ineligible due to pack-years less than 30 or quit smoking more than 15 years ago	7040	7709	8204	10847
Excluded: diagnosed with lung cancer >1 year ago	21	30	31	44
Excluded: newly diagnosed with lung cancer in the past year	10	8	15	31
Denominator: patients eligible for LCS	1104	1219	1255	1865

© 2024 Kukhareva PV et al. *JAMA Network Open.*

e-Table S2. Overall patient population demographics.

Characteristics	Overall (n=1865)
Age, median (IQR)	64.0 (60.0, 70.0)
Sex assigned at birth, N(%)	
Female	759 (40.7%)
Male	1106 (59.3%)
Race/ethnicity, N(%)	
Black/AA, Non-Hispanic	36 (1.9%)
Hispanic, any race	98 (5.3%)
White, Non-Hispanic	1574 (84.4%)
Other	157 (8.4%)

© 2024 Kukhareva PV et al. *JAMA Network Open.*

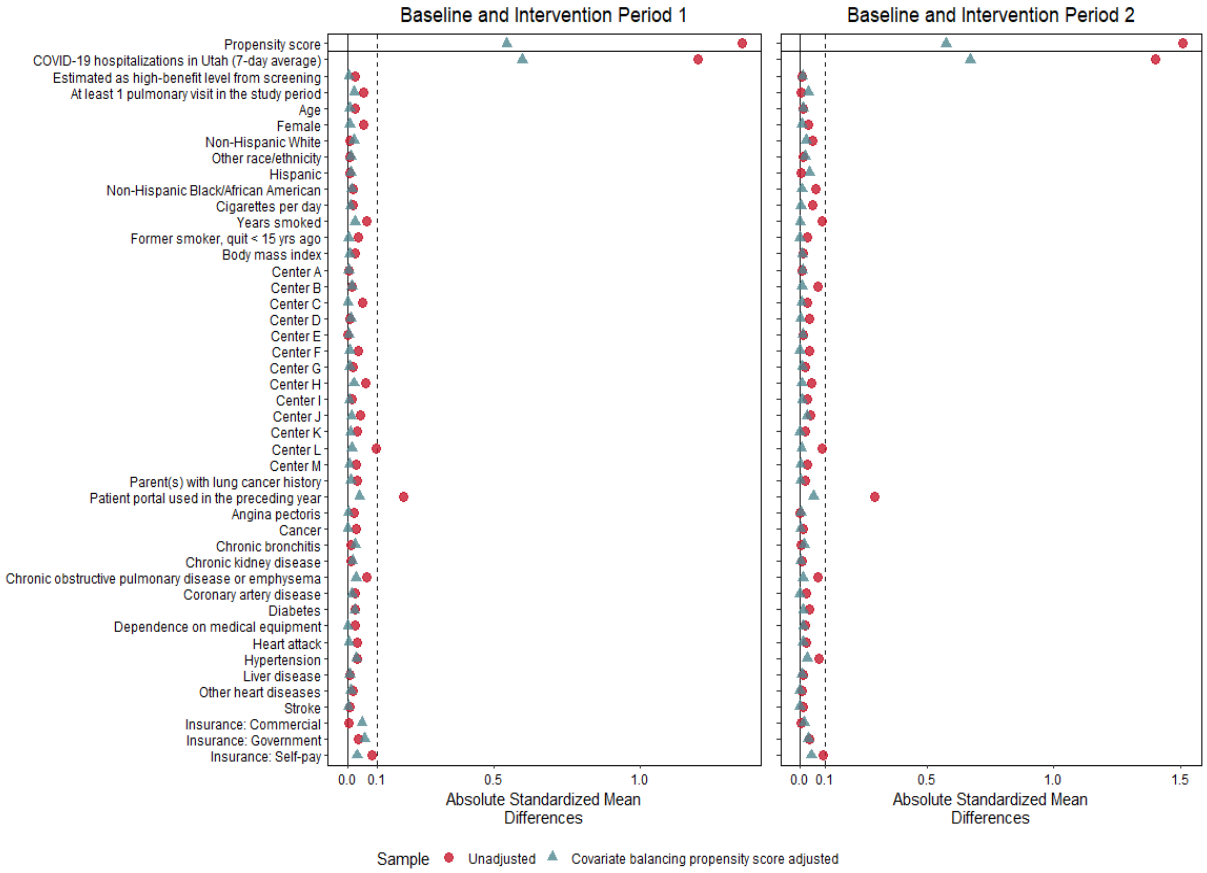

e-Figure S2. Covariate Balancing Propensity Score (CBPS) Model and Improvement in Covariates Balance for Baseline and Intervention Periods 1 and 2

Note: We calculated the absolute standardized mean difference to measure the effect size of differences between periods with and without adjustment by the CBPS approach. E-Figure S2 summarizes the covariate balance between baseline period and intervention phase 1, and the covariate balance between baseline period and intervention phase 2, separately. The solid vertical line at 0 represents no difference between the groups, and the dashed line at 0.1 serves as a threshold indicating an acceptable level of covariate balance. The plot shows that all the covariates were balanced after CBPS adjustment except for COVID-19 hospitalization.

© 2024 Kukhareva PV et al. *JAMA Network Open.*
