# Supplementary material for: Lung Cancer Screening Before and After a Multifaceted Electronic Health Record Intervention: A Nonrandomized Controlled Trial
Source: JAMA Netw Open. 2024 Jun 7;7(6):e2415383. doi: 10.1001/jamanetworkopen.2024.15383 (PMC11161845; doi:10.1001/jamanetworkopen.2024.15383)
Supplement: Supplement 3. — Data Sharing Statement [file jamanetwopen-e2415383-s003.pdf]

Data Sharing Statement

Kukhareva. Lung Cancer Screening Before and After a Multifaceted Electronic Health Record Intervention: A Nonrandomized Controlled Trial. *JAMA Netw Open*. Published online June 7, 2024. doi:10.1001/jamanetworkopen.2024.15383

Data
Data available: No

Additional Information
Explanation for why data not available: The data underlying this article cannot be shared publicly due to UUH policies regarding the privacy of patients and the protection of sensitive patient health information.
